# Enhanced mGluR1 function causes motor deficits and region-specific Purkinje cell dysfunction

**DOI:** 10.1093/brain/awaf477

**Published:** 2026-01-12

**Authors:** Mohamed F Ibrahim, Sevda Boyanova, Yin Chun Cheng, Clemence Ligneul, Rasneer S Bains, Tiffany C Johnpulle, Jason P Lerch, Edward O Mann, Peter L Oliver, Esther B E Becker

**Affiliations:** Nuffield Department of Clinical Neurosciences, University of Oxford, Oxford OX3 9DU, UK; Kavli Institute for Nanoscience Discovery, University of Oxford, Oxford OX1 3QU, UK; Nuffield Department of Clinical Neurosciences, University of Oxford, Oxford OX3 9DU, UK; Kavli Institute for Nanoscience Discovery, University of Oxford, Oxford OX1 3QU, UK; Nuffield Department of Clinical Neurosciences, University of Oxford, Oxford OX3 9DU, UK; Kavli Institute for Nanoscience Discovery, University of Oxford, Oxford OX1 3QU, UK; Wellcome Centre for Integrative Neuroimaging, FMRIB, Nuffield Department of Clinical Neurosciences, University of Oxford, Oxford OX3 9DU, UK; Mary Lyon Centre at MRC Harwell, Didcot OX11 0RD, UK; Nuffield Department of Clinical Neurosciences, University of Oxford, Oxford OX3 9DU, UK; Kavli Institute for Nanoscience Discovery, University of Oxford, Oxford OX1 3QU, UK; Wellcome Centre for Integrative Neuroimaging, FMRIB, Nuffield Department of Clinical Neurosciences, University of Oxford, Oxford OX3 9DU, UK; Department of Medical Biophysics, University of Toronto, Toronto, ON, M5G 2C4, Canada; Department of Physiology, Anatomy and Genetics, University of Oxford, Oxford OX1 3PT, UK; Mammalian Genetics Unit, MRC Harwell Institute, Didcot OX11 0RD, UK; Nuffield Department of Clinical Neurosciences, University of Oxford, Oxford OX3 9DU, UK; Kavli Institute for Nanoscience Discovery, University of Oxford, Oxford OX1 3QU, UK

**Keywords:** cerebellum, Grm1, mGluR1, Purkinje cell, ataxia, selective vulnerability

## Abstract

Spinocerebellar ataxias (SCAs) are autosomal dominantly inherited neurodegenerative disorders with no effective treatment. Aberrant signalling through the metabotropic glutamate receptor (mGluR1) has been implicated in several SCAs. However, whether disease is caused through decreased or increased mGluR1 signalling remains controversial.

Here, we generate the first mouse model of enhanced mGluR1 function by introducing a patient gain-of-function mutation (p.Y792C) that causes SCA44 in the metabotropic glutamate receptor 1 (*Grm1*) gene. *Grm1* mutant mice recapitulate key pathophysiological aspects of SCA, including progressive motor deficits, altered climbing fibre innervation and perturbed Purkinje cell (PC) spontaneous activity. We report that changes in synaptic innervation and intrinsic PC activity upon overactive mGluR1 signalling manifest in a lobule- and disease-stage-specific manner.

Our findings demonstrate that enhanced mGluR1 function is a direct and specific driver of PC dysfunction and pathology and provide a mechanism for understanding the selective vulnerability of different PC populations in SCA.

## Teaser

Aberrant signalling through metabotropic glutamate receptor 1 (mGluR1) has been implicated in several forms of spinocerebellar ataxia. Ibrahim *et al.* show that introducing a gain-of-function mutation into the mouse mGluR1 (*Grm1*) gene disrupts spontaneous Purkinje cell activity and recapitulates key motor deficits.

## Introduction

The spinocerebellar ataxias (SCAs) are a genetically and clinically complex group of autosomal dominant neurodegenerative diseases that primarily affect the cerebellum and its associated pathways.^1,2^ SCAs are characterized by a progressive loss of balance and coordination accompanied by slurred speech, difficulties in swallowing and oculomotor abnormalities. SCAs are heterogeneous, and to date more than 40 genetically defined subtypes have been identified. The overall rarity of SCAs, together with the diverse genes and implicated pathways that cause the disease, renders the feasibility of finding therapeutics for individual subtypes challenging.^3^ Therefore, a deeper understanding of the disease mechanisms that might be shared by distinct SCA subtypes is critical to advancing treatment options.

Emerging evidence indicates that aberrant signalling through the metabotropic glutamate receptor type 1 (mGluR1) is a central mechanism in many SCAs.^4,5^ mGluR1 is highly expressed in Purkinje cells (PCs) and plays a crucial role in the development, synaptic wiring, spontaneous activity and plasticity of PCs.^5-8^ Intriguingly, both reduced and increased mGluR1 function have been implicated in cerebellar ataxia in human patients and genetically engineered mouse models. Autoantibodies against mGluR1 and downstream signalling molecules have been identified in autoimmune ataxias,^9^ and homozygous loss-of-function mutations in the *GRM1* gene encoding mGluR1 are associated with autosomal recessive spinocerebellar ataxia 13 (SCAR13).^10,11^ Downregulation of mGluR1 and associated signalling molecules has been reported in some mouse models of SCAs including SCA1, SCA2 and SCA3.^12-16^ However, it remains controversial whether these changes are an early, pathogenic mechanism in SCA or whether they represent a later, potentially compensatory consequence to maintain calcium homeostasis. In support of the latter, prolonged and thus enhanced mGluR1 currents were observed in the conditional SCA1 *ATXN1[82Q]* mouse model, which has a more moderate phenotype and likely represents an early disease stage of SCA1.^17^ mGluR1 signalling was also found to be greatly amplified early in disease in a mouse model of SCA2.^18^ In support of the hypothesis that overactive mGluR1 signalling causes cerebellar ataxia, gain-of-function (GOF) mutations in the TRPC3 cation channel that is activated by mGluR1 cause cerebellar ataxia in the *Moonwalker* (*Mwk*) mouse mutant and patients with SCA41.^19,20^ Similarly, in a mouse model of SCA14 mutant protein kinase C gamma (PKCγ) fails to inhibit TRPC3 and, as a consequence, mGluR1-mediated currents are increased.^21^ Based on these and other studies, it has been postulated that a positive feedback loop between enhanced mGluR1 signalling and elevated calcium levels in PCs might be a common driver of PC dysfunction and loss in SCA.^18^ However, so far, evidence has been lacking on whether enhanced mGluR1 function can directly cause PC dysfunction and disease.

In this study, we present a mouse model of enhanced mGluR1 function that allowed us to investigate for the first time the direct impact of altered mGluR1 activity on PC and cerebellar function. We previously reported the first dominant mutations in the *GRM1* gene causing SCA44 in two families with adult-onset cerebellar ataxia and degeneration.^22^ Using an mGluR1 reporter assay in heterologous cell lines, we showed that the SCA44 patient *GRM1* mutations (p.Y262C and p.Y792C) behaved as GOF mutations in vitro.^22^ This is consistent with a recent pharmacological study that demonstrated enhanced constitutive activity of the SCA44 p.Y792C mutation in calcium mobilization studies in HEK293 cells.^23^ While these findings support a role for enhanced mGluR1 activity in SCA44, the effect of the GOF *GRM1* mutations on the cerebellum remained unexplored, and thus the pathophysiological mechanisms underlying SCA44 are not well understood. We therefore developed a *Grm1* mutant mouse model harbouring the SCA44 p.Y792C mutation and demonstrate that this mutation results in enhanced mGluR1 signalling in PCs. Behaviourally, heterozygous *Grm1*^Y792C/+^ mutant mice recapitulate the late-onset ataxic phenotype observed in human SCA44 patients, whereas homozygous *Grm1*^Y792C/Y792C^ mutant mice develop progressive cerebellar ataxia from 3 months of age. Moreover, *Grm1* mutants display altered climbing fibre (CF)-PC synaptic connectivity and aberrant PC activity, which are both key pathological hallmarks of SCAs. Notably, we also show that the observed structural and functional deficits are region- and disease-stage-dependent. Together, our study demonstrates that enhanced mGluR1 function is a direct driver of selective PC dysfunction leading to cerebellar ataxia.

## Materials and methods

### Animals and husbandry

All procedures and experiments were carried out in accordance with the Animals (Scientific Procedures) Act 1986, UK, amendment Regulations 2012 (SI 4 2012/3039). All mice were group-housed and kept under a 12-h day/night cycle with food and water available *ad libitum* in a controlled environment maintained at a temperature of 22 ± 2°C with a humidity level of 55% ± 10%.

### Behavioural testing

Two cohorts of male mice were used for the longitudinal behavioural testing at 3, 6, 12 and 18 months of age (for animal numbers at each age, see Supplementary Table 1). Behavioural tests were performed in the same order: (i) open field; (ii) light/dark box; (iii) elevated zero maze; (iv) forced alteration Y-maze; (v) Locotronic gait analysis; (vi) MouseWalker gait analysis; and (vii) balance beam. The balance beam testing began only at 6 months of age. A second cohort of naive male mice was used for the balance beam testing at 3 months of age. Fear conditioning and prepulse inhibition testing were carried out only at 6 months of age. Experimentally naive female mice were used for the accelerating Rotarod testing, balance beam test, Locotronic, and MouseWalker gait analysis at 3 and 12 months of age. All behavioural experiments and related data analysis was done blinded. The order of animals for each behavioural test was randomized. For details on behavioural tests, see the Supplementary material.

### Immunohistochemistry and imaging

Mice were anaesthetized and transcardially perfused with 10% neutral buffered formalin (NBF). Cerebella were removed and postfixed with 10% NBF at 4°C, then cryoprotected with 30% sucrose in PBS at 4°C. Fixed tissues were embedded in optimal cutting temperature (OCT) medium (Thermo Scientific) and cut on a cryostat into 30 µm thick parasagittal sections mounted on SuperFrost Plus slides. Slides were washed in PBS for 10 min, incubated in 0.1 M glycine for 30 min in a humidified chamber, incubated in blocking buffer (10% normal goat serum, 0.3% Triton-X 100 in PBS) for 1 h at room temperature (RT) and incubated overnight at 4°C with the following primary antibodies in blocking buffer: anti-vGluT1 (1:500; Synaptic Systems; 135303), anti-vGluT2 (1:500, Synaptic Systems; 135403), anti-Calbindin D28 K (1:500; Synaptic Systems; 214005), anti-mGluR1α (1:1000; Nittobo Medical; mGluR1α-GP-Af660). After incubation, slides were washed in blocking buffer three times, 10 min each, and incubated for 3 h at RT with the following secondary antibodies: AlexaFluor 488 goat anti-rabbit (1:500, Invitrogen, A32731) and AlexaFluor 594 goat anti-guinea pig (1:500, Invitrogen, A-11076). Slides were washed three times, 10 min each, in blocking buffer and once in PBS, before mounting in Vectashield mounting media (Vector Laboratories, H-1200). Images were acquired using an Axiovision fluorescent microscope (Zeiss) with 2.5× and 20× objective lenses. All quantifications were performed with 20× images using the ‘measure’ function of Fiji/ImageJ software (version 2.9.0/1.53). At least 2–3 sections were quantified for each lobule (III, V and X) per mouse. Molecular layer thickness was measured as the distance from the top of the PC soma to the top of the dendritic tree. CF territory was expressed as the height of the VGluT2-positive CF terminals (distance from the top of the PC soma to the highest VGluT2 puncta in the molecular layer) divided by the molecular layer thickness. Density of PCs were measured by dividing the number of Calbindin-positive PCs by the length of each section in the lobules. Calbindin-positive PC somata were selected using the ‘freehand selection’ function of Fiji/ImageJ and their areas were measured.

### Cerebellar slice preparation for *ex vivo* electrophysiology

Mice were anaesthetized with 4% isoflurane and swiftly decapitated. Old mice (15–16 months) were anaesthetized with an intraperitoneal injection of Phenobarbitone overdose, after which rapid transcardial perfusion with ice-cold *N*-methy-D-glucamine (NMDG) artificial CSF (aCSF) cutting solution (Supplementary material) was performed before decapitation. For cell-attached recording experiments, extracted brains were chilled and parasagittal (250 µm) cerebellar vermal slices were cut in ice-cold NMDG aCSF cutting solution. Slices were recovered in a hot NMDG aCSF cutting solution (35°C) for 10–12 min, after which slices were kept in HEPES aCSF at RT until recording. For whole-cell mGluR1 recordings, brains were chilled and parasagittal (250 µm) cerebellar vermal slices were cut with ice-cold sucrose cutting aCSF. Slices were recovered for 30 min in hot normal aCSF (35°C) and cooled to RT before recording.

### Extracellular cell-attached recordings

Extracellular cell-attached recordings were performed at 30°C–31°C in aCSF constantly bubbled with carbogen. Recording glass patch pipettes (World Precision instruments), of 4–6 MΩ resistance (PC-100, Narshinge) filled with aCSF were used. PC spontaneous activity was recorded for 10 min, with recordings between 6–9 min being used for analysis. The interspike interval (ISI) was measured and the mean firing rate (inverse of the mean ISI), coefficient of variation (CV) and the local coefficient of variation (CV2) were quantified using the following formulas:


(1) $$\mathrm{CV}{=}^{\sigma }(\mathrm{ISI}){/}^{\mu }(\mathrm{ISI})$$

(2) $$\mathrm{CV}2=\mathrm{mean}\left[2\times \frac{\left|\mathrm{IS}I_{n}-\mathrm{IS}I_{n\text{-}1}\right|}{(\mathrm{IS}I_{n}+1+\mathrm{IS}I_{n})}\right]$$

### Whole-cell electrophysiology

For whole-cell voltage-clamp recordings (5–7-week-old mice), recording pipettes were filled with internal solution containing (in mM): 122 K-methane sulphonate, 9 NaCl, 9 HEPES, 0.036 CaCl_2_, 1.62 MgCl_2_, 0.18 EGTA, 4 Mg-ATP, 0.3 guanosine 5'-triphosphate tris salt, 14 Tris-creatine (all Sigma-Aldrich). Cells were held at −70 mV, and series resistance compensation of up to 70% was made when possible. We excluded recordings with a series resistance greater than 30 MΩ. PF stimulation was adjusted to evoke an AMPA-mediated fast excitatory postsynaptic currents (EPSCs) with an amplitude of 450–600 pA in the presence of GABA(A) receptor antagonist Gabazine (15 µM; Hellobio; HB0901). Parallel fibre (PF)-mediated mGluR1 slow EPSC were evoked by stimulation of PFs (200 Hz; 200-μs duration; 2-sweep average) with 2, 5, 10 or 20 pulses, and measured in the presence of AMPA/kainate receptor blocker NBQX (20 µM; Hellobio; HB0442) and glutamate transporter blocker DL-threo-β-benzyloxyaspartic acid (TBOA) (100 µM; Hellobio; HB0258). All electrophysiology data were acquired using a Multiclamp 700B amplifier (Molecular Devices, LLC.), filtered at 4 kHz online, digitized at 20 kHz with ICT18 (Heka Instrument, Inc.). Data acquisition and analysis were carried out using custom scripts in Igor pro software (WaveMetrics Inc., Portland, OR, USA).

### Calcium imaging

For PC calcium imaging (3–4-week-old mice), cells were whole-cell patched and filled with an internal solution containing (in mM): 130 potassium gluconate, 4 KCl, 10 HEPES, 10 sodium creatine phosphate, 0.2 EGTA, 4 MgATP, 0.4 NaGTP, pH titrated to 7.3–7.4 with KOH (all Sigma-Aldrich) and Ca^2+^ indicator Oregon Green BAPTA-1 (OGB-1) (200 μM; Thermo Fisher Scientific; 06806) for at least 25 min. For the local application of 3,5-dihydroxyphenylglycine (DHPG) on PC dendrites, DHPG was pressure ejected (200 μM; 20 ms; 10 psi) using a Picospritzer III (Parker Hannifin Corporation). Wide-field images were acquired with an Olympus BX51WI wide-field microscope equipped with a 40×/0.8 numerical aperture water objective (Olympus), OptoLED Lite system (Cairn Research Ltd.) and an optiMOS sCMOS camera (QImaging) with 50 ms/frame and a 10-ms inter-frame interval for 400 frames.

Analysis was performed using custom MATLAB (R2023b) scripts. Active pixels were defined as those for which the signal exceeded 3 × standard deviation above the baseline mean (frames 50–100) for 25 frames around the peak. The mean signal across all active pixels was bleach-corrected by fitting an exponential (50–150 frames), the fitted decay was subtracted, and the response was quantified as the fractional change in fluorescence relative to the baseline mean (50–150 frames; ΔF/F). Peak amplitude and area under the curve were calculated.

### Statistical analysis

For behavioural, electrophysiological, immunohistochemical and western blotting data, data were tested for normality using the Shapiro–Wilk normality test. We used parametric (*t*-test or one-/two-way ANOVA) for normally distributed data and non-parametric (Kruskal–Wallis or Mann–Whitney) statistical tests for data with non-normal distribution, followed by pairwise multiple comparison tests as indicated in the figure legends. Data are reported as mean ± standard error of the mean (SEM). All figures and statistical analysis were computed using Prism v.10 (GraphPad Software Inc., CA, USA). All statistical data are available in the Supplementary material.

Additional methodological details are available in the Supplementary material.

## Results

### Introduction of a SCA44 patient mutation into mouse *Grm1* results in enhanced mGluR1 activity in Purkinje cells

**Figure 1:**
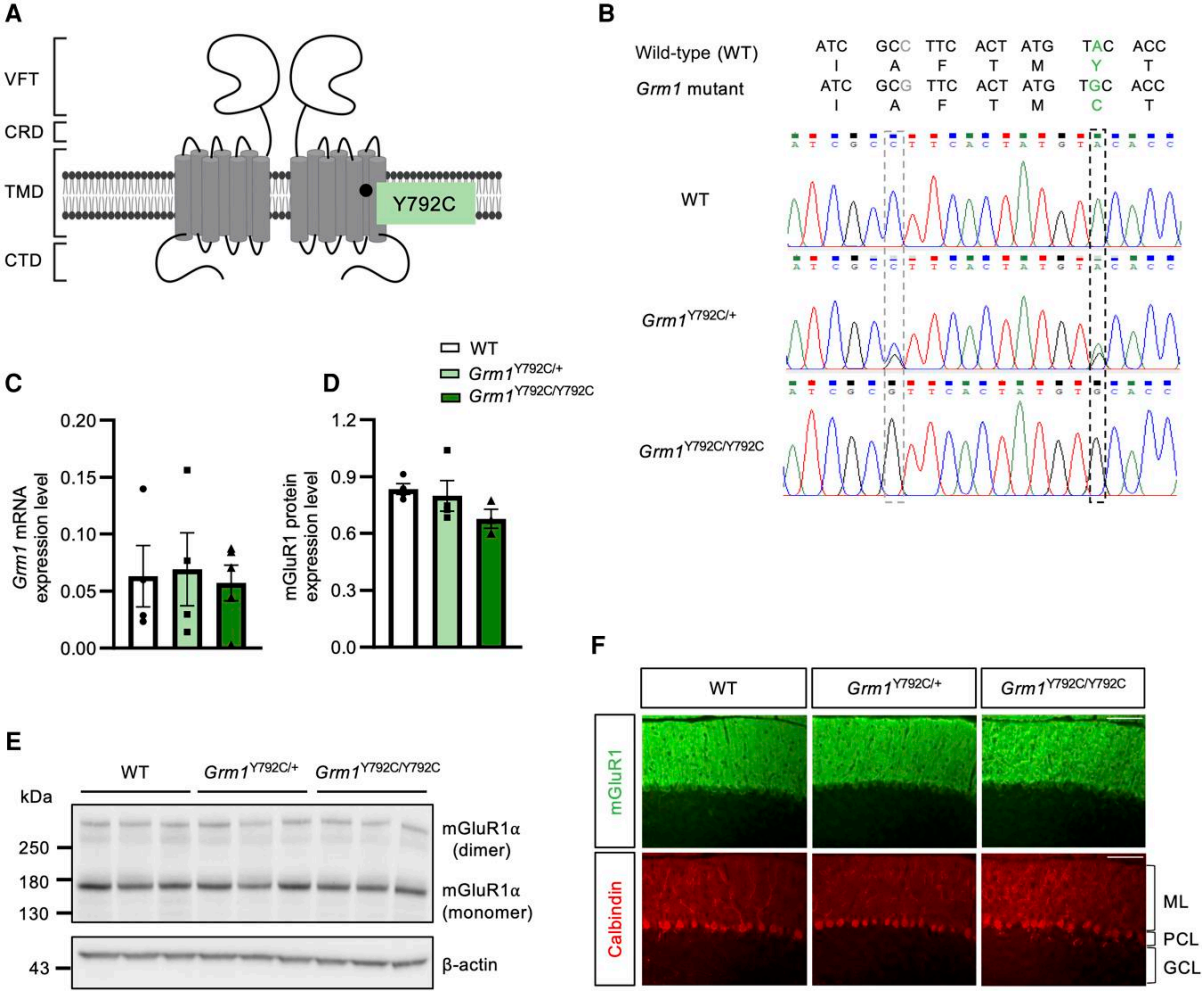
**Characterization of metabotropic glutamate receptor (mGluR1) expression in *Grm1* mutant cerebellum.** (A) Schematic representation of the SCA44 p.Y792C mutation within the mGluR1 receptor. VFT = Venus fly trap domain; CRD = cysteine-rich domain; TMD = transmembrane domain; CTD = intracellular C-terminal domain. Created in BioRender. Becker, E. (2025) https://BioRender.com/um345pp. **(B)** Sanger sequencing confirms successful Cas9-mediated base-pair editing. In addition to the SCA44 point mutation (A>C; p.Tyr792Cys), a silent base pair substitution (C>G; p.Ala782Ala) was created in the nearby protospacer adjacent motif (PAM) site to prevent re-cutting by the Cas9 enzyme. (C) Quantification of relative *Grm1* mRNA expression levels in 3-month-old wild-type (WT) and *Grm1* mutant mouse cerebellum by reverse transcription quantitative PCR. Expression calculated as 2^−ΔCT^, relative to housekeeper gene *Gapdh.* Three technical replicates were included in all experiments. Data-points represent individual animals. WT versus *Grm1*^Y792C/+^: *P* > 0.9999, WT versus *Grm1*^Y792C/Y792C^: *P* > 0.9999, *Grm1*^Y792C/+^ versus *Grm1*^Y792C/Y792C^: *P* > 0.9999. *n* = 4–5 animals per genotype. One-way ANOVA followed by Bonferroni’s multiple comparison test. Error bars represent standard error of the mean (SEM). (D) Quantification of relative mGluR1 protein expression levels in 3-month-old WT and *Grm1* mutant cerebellum. Protein levels are normalized to β-actin. Data-points represent individual animals. WT versus *Grm1*^Y792C/+^: *P* > 0.9999, WT versus *Grm1*^Y792C/Y792C^: *P* = 0.3237, *Grm1*^Y792C/+^ versus *Grm1*^Y792C/Y792C^: *P* = 0.6007. *n* = 3–4 animals per genotype. One-way ANOVA followed by Bonferroni's multiple comparisons. Error bars represent SEM. For full statistical data, see Supplementary Table 2. (E) Representative immunoblotting images showing expression of mGluR1 in the cerebellum of 3-month-old WT and *Grm1* mutant littermates (*n* = 3 animals per genotype). β-actin was used as loading control. Uncropped images with molecular weight markers are available in Supplementary Fig. 7. (F) Representative immunostaining against mGluR1 (green) and Calbindin (red) in 3-month-old WT and *Grm1* mutant cerebellum. ML = molecular layer; PCL = Purkinje cell layer; GCL = granule cell layer. Scale bar = 100 μm.

To investigate the pathophysiological mechanisms underlying SCA44 and, more broadly, to understand the role of enhanced mGluR1 signalling in cerebellar function and disease, we generated a mouse model harbouring the mGluR1 p.Y792C SCA44 patient GOF mutation.^22^ Tyr792 is located within the sixth helix transmembrane domain of mGluR1 (Fig. 1A), a critical region for receptor activation.^24^ To generate the SCA44 patient mutation in the mouse, we employed clustered regularly interspaced short palindromic repeats (CRISPR)-Cas9-mediated gene editing and introduced an A-to-G base pair substitution in the endogenous mouse *Grm1* gene, resulting in the mGluR1 p.Y792C missense mutation. The successful generation of both heterozygous (*Grm1*^Y792C/+^) and homozygous (*Grm1*^Y792C/Y792C^) mutant animals was validated using Sanger sequencing (Fig. 1B).

We first assessed whether the introduced missense mutation would affect mGluR1 expression in the cerebellum by quantifying *Grm1* mRNA and mGluR1 protein levels. We did not observe any significant changes in *Grm1* mRNA expression (Fig. 1C) or mGluR1 protein levels in the cerebellum across different ages (Fig. 1D and E; Supplementary Fig. 1) in either heterozygous or homozygous *Grm1* mutant mice compared to wild-type (WT) littermates. The mGluR1 receptor is highly expressed in PC dendrites,^25^ and we found that the dendritic localization of mGluR1 in PCs was not altered upon the introduction of the Y792C mutation (Fig. 1F).

**Figure 2:**
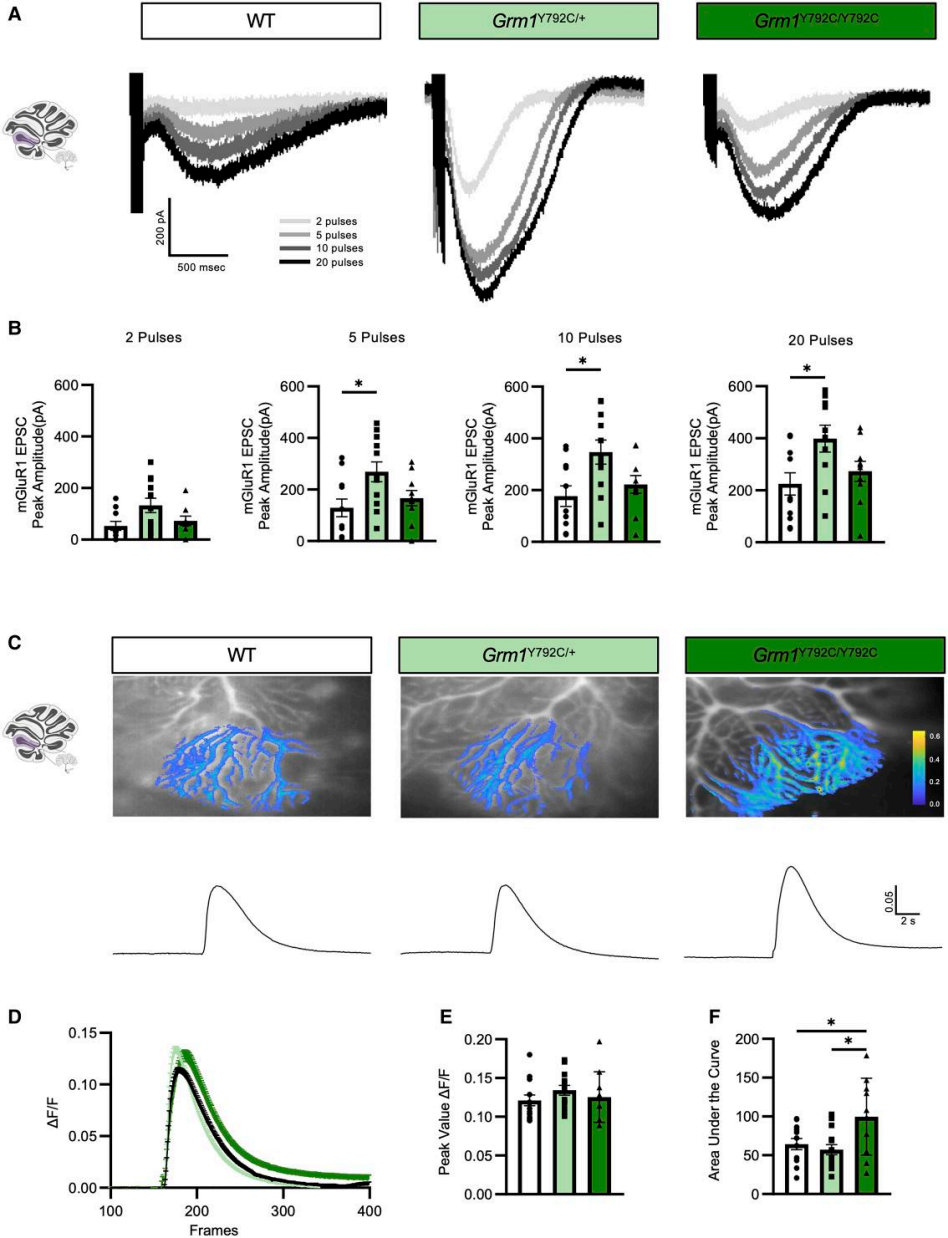
**Characterization of metabotropic glutamate receptor (mGluR1)-mediated synaptic currents and calcium transients in *Grm1* mutant cerebellum.** (A) Representative traces of parallel fibre-evoked slow mGluR1 excitatory postsynaptic currents (EPSC)s in wild-type (WT) and *Grm1* mutant Purkinje cells (PCs) in lobule III of 5–7-week-old mice measured in extracellular solution containing antagonists for AMPA/kainate receptors, GABA(A) receptors and glutamate transporters. (B) Quantification of average-peak slow mGluR1 EPSC for increasing pulses (2, 5, 10 and 20 pulses) of 200-Hz stimulations. *n* = 9–12 cells per genotype. 2 Pulses: WT versus *Grm1*^Y792C/+^: *P* = 0.0711, WT versus *Grm1*^Y792C/Y792C^: *P* > 0.9999; *Grm1*^Y792C/+^ versus *Grm1*^Y792C/Y792C^: *P* > 0.5149; 5 Pulses: WT versus *Grm1*^Y792C/+^: *P* = 0.0359, WT versus *Grm1*^Y792C/Y792C^: *P* > 0.9999, *Grm1*^Y792C/+^ versus *Grm1*^Y792C/Y792C^: *P* = 0.3142; 10 Pulses: WT versus *Grm1*^Y792C/+^: *P* = 0.0155, WT versus *Grm1*^Y792C/Y792C^: *P* = 0.7328, *Grm1*^Y792C/+^ versus *Grm1*^Y792C/Y792C^: *P* = 0.0998; 20 Pulses: WT versus *Grm1*^Y792C/+^: *P* = 0.0252, WT versus *Grm1*^Y792C/Y792C^: *P* = 0.7245; *Grm1*^Y792C/+^ versus *Grm1*^Y792C/Y792C^: *P* = 0.1397. (C) Representative images of changes in calcium fluorescence in PC dendrites in lobule III of 3–4-week-old mice in response to local dendritic puff application of the group I mGluR-agonist 3,5-dihydroxyphenylglycine hydrate (DHPG). (D) Line plot of the mean traces of normalized Oregon Green BAPTA-1 (OGB-1) fluorescence responses (ΔF/F) in PC dendrites. (E) Bar graphs showing the peak value (ΔF/F) quantified from **D**. WT versus *Grm1*^Y792C/+^: *P* = 0.4176, WT versus *Grm1*^Y792C/Y792C^: *P* = 0.9256, *Grm1*^Y792C/+^ versus *Grm1*^Y792C/Y792C^: *P* = 0.7064. (F) Area under the curve quantified from **D**. WT versus *Grm1*^Y792C/+^: *P* = 0.8366, WT versus *Grm1*^Y792C/Y792C^: *P* = 0.0436, *Grm1*^Y792C/+^ versus *Grm1*^Y792C/Y792C^: *P* = 0.0100. *n* = 10–15 cells per genotype. Statistical significance was determined by one-way ANOVA followed by Tukey’s multiple comparison test or Kruskal–Wallis test followed by Dunn’s multiple comparison test. Error bars represent standard error of the mean. *P < 0.05. For full statistical data, see Supplementary Table 2. Cerebellar icons created in BioRender. Becker, E. (2025) https://BioRender.com/um345pp.

Next, we examined the functional impact of the Y792C mutation on mGluR1-mediated signalling. mGluR1 activation produces a complex postsynaptic response in PCs consisting of two distinct signalling components: a slow excitatory postsynaptic current (EPSC) at the parallel fibre (PF)-PC synapse^26^ and local inositol 14,5-triphosphate (IP_3_) receptor-mediated calcium release from internal stores.^27^ We recorded slow EPSCs in adult PCs from WT and *Grm1* mutants in response to an increased number of pulses delivered to the innervating PFs. *Grm1*^Y792C/+^ PCs exhibited significantly larger slow EPSCs than WT PCs (Fig. 2A and B). In *Grm1*^Y792C/Y792C^ PCs, slow EPSCs were larger but not significantly different from WT PCs. To study mGluR1-mediated dendritic calcium changes in PCs, we applied the group I mGluR agonist, DHPG, locally to distal dendrites. There were no significant differences in the peak of dendritic calcium signals between the genotypes (Fig. 2C–E). However, the area under the curve was significantly larger in *Grm1*^Y792C/Y792C^ PCs compared with both WT and *Grm1*^Y792C/+^ mice (Fig. 2C–F), likely reflecting a more prolonged response. Together, these results demonstrate that the p.Y792C mutation does not affect the localization of mGluR1 but causes enhanced mGluR1 function in PCs, with genotype-dependent heterogeneity across the two components of the mGluR1 signalling pathway.

### *Grm1* mutant mice display a progressive decline in motor coordination

To investigate whether the introduced SCA44 mutation and resulting enhanced mGluR1 function affects motor function, we carried out a longitudinal behavioural study in *Grm1* mutant animals compared with WT littermates from 3 to 18 months of age, representing young adulthood until old age. We assessed both heterozygous and homozygous *Grm1* mutants, as we predicted that the latter could serve as an accelerated disease model.^28^

**Figure 3:**
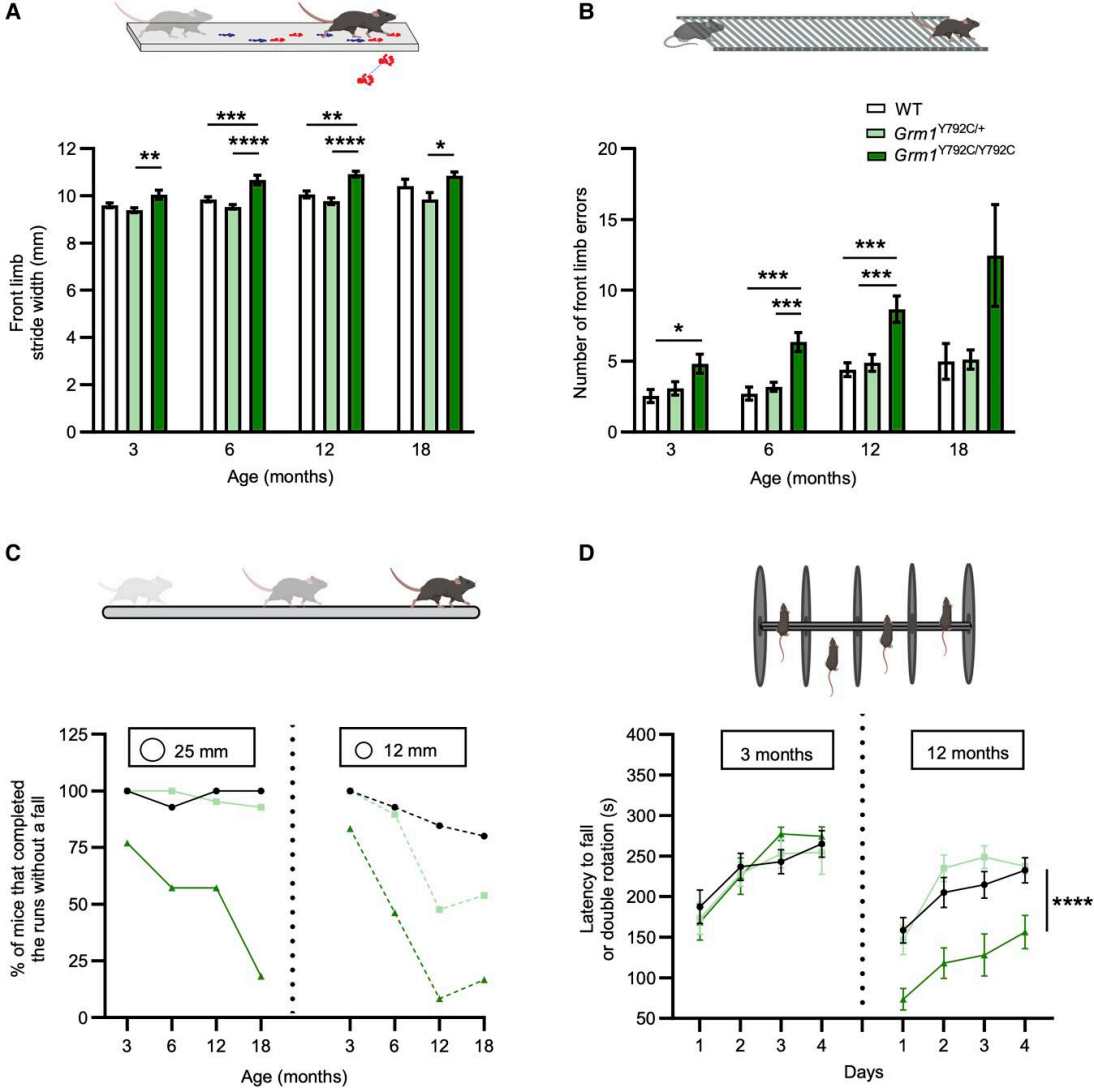
***Grm1* mutant mice develop progressive loss of motor performance and coordination.** (A) Quantification of the front limb stride width, i.e. the distance between the right and left front limb placement, assessed using the MouseWalker system. 3 months: wild-type (WT) versus *Grm1*^Y792C/+^: *P* = 0.4787, WT versus *Grm1*^Y792C/Y792C^: *P* = 0.0707, *Grm1*^Y792C/+^ versus *Grm1*^Y792C/Y792C^: *P* = 0.0020; 6 months: WT versus *Grm1*^Y792C/+^: *P* = 0.2273, WT versus *Grm1*^Y792C/Y792C^: *P* = 0.0008, *Grm1*^Y792C/+^ versus *Grm1*^Y792C/Y792C^: *P* < 0.0001; 12 months: WT versus *Grm1*^Y792C/+^: *P* = 0.3509, WT versus *Grm1*^Y792C/Y792C^: *P* = 0.0012, *Grm1*^Y792C/+^ versus *Grm1*^Y792C/Y792C^: *P* < 0.0001; 18 months: WT versus *Grm1*^Y792C/+^: *P* = 0.4292, WT versus *Grm1*^Y792C/Y792C^: *P* = 0.6214, *Grm1*^Y792C/+^ versus *Grm1*^Y792C/Y792C^: *P* = 0.0201. Statistical significance was determined by one-way ANOVA followed by Tukey’s multiple comparison test. For *n*-numbers, see Supplementary Table 1. (B) Quantification of the number of front limb errors using the Locotronic apparatus. 3 months: WT versus *Grm1*^Y792C/+^: *P* > 0.9999, WT versus *Grm1*^Y792C/Y792C^: *P* = 0.0468, *Grm1*^Y792C/+^ versus *Grm1*^Y792C/Y792C^: *P* = 0.0992; 6 months: WT versus *Grm1*^Y792C/+^: *P* > 0.9999, WT versus *Grm1*^Y792C/Y792C^, *P* = 0.0002, *Grm1*^Y792C/+^ versus *Grm1*^Y792C/Y792C^: *P* = 0.0007; 12 months: WT versus *Grm1*^Y792C/+^: *P* = 0.8573, WT versus *Grm1*^Y792C/Y792C^: *P* = 0.0003, *Grm1*^Y792C/+^ versus *Grm1*^Y792C/Y792C^: *P* = 0.0008; 18 months: WT versus *Grm1*^Y792C/+^: *P* > 0.9999, WT versus *Grm1*^Y792C/Y792C^: *P* = 0.0093, *Grm1*^Y792C/+^ versus *Grm1*^Y792C/Y792C^: *P* = 0.0526. Statistical significance was determined by one-way ANOVA followed by Tukey’s multiple comparison test or Kruskal–Wallis test followed by Dunn’s multiple comparison test. For *n*-numbers, see Supplementary Table 1. (C) Percentage of WT and *Grm1* mutant mice that have completed runs on the balance beam without a fall assessed on a wide (25 mm) and narrow (12.5 mm) beam. For *n*-numbers, see Supplementary Table 1. (D) Motor coordination represented as latency to fall or double rotation on an accelerating Rotarod of naive female WT and *Grm1* mutant mice assessed at 3 and 12 months of age. 3 months: Genotype: *F*(2,27) = 0.1120, *P* = 0.8495; Day: *F*(2.007,54.20) = 25.34, *P* < 0.0001; 12 months: Genotype: *F*(2,30) = 15.66, *P* < 0.0001; Day: *F*(2.642,79.27) = 25.38, *P* < 0.0001. Statistical significance was determined by two-way ANOVA followed by Tukey’s *post hoc* test. Error bars represent standard error of the mean. *P < 0.05, **P < 0.01, ***P < 0.001, ****P < 0.0001. For *n*-numbers, see Supplementary Table 1. For full statistical data, see Supplementary Table 2. Behavioural test icons created in BioRender. Becker, E. (2025) https://BioRender.com/um345pp.

First, we assessed the gait of the mutant mice using the MouseWalker system that allows the high-resolution tracking of locomotion features such as limb positioning during walking.^29^ Starting at 3 months of age, *Grm1*^Y792C/Y792C^ mice had a significantly larger front limb stride, which became worse with increasing age (Fig. 3A). No statistical difference was detected at 18 months of age, likely reflecting a reduction in the experimental cohort size due to ageing. When we examined motor impairments during walking across a horizontal ladder using the Locotronic apparatus, *Grm1*^Y792C/Y792C^ mice displayed increased front limb errors from 3 months of age, which worsened progressively with increasing age of the tested animals (Fig. 3B). *Grm1*^Y792C/+^ animals did not show any impairments during the testing period.

We also assessed motor coordination and balance of the animals using a horizontal balance beam walking task with two beams of varying diameter. *Grm1*^Y792C/+^ mice performed to a similar level to WT mice on the wider beam at all ages of testing, with most animals able to traverse the beam without a fall. In contrast, the percentage of *Grm1*^Y792C/Y792C^ mice that successfully crossed the beam without a fall decreased markedly with increasing age (Fig. 3C). On the narrower beam, *Grm1*^Y792C/Y792C^ mice showed impairments as early as 3 months of age, while deficits in *Grm1*^Y792C/+^ mice were evident from 12 months of age.

To investigate if there was any sex difference in the impact of motor coordination, we also examined gait and balance in a cohort of naive 12-month-old female mutant mice. Similar to males, female *Grm1*^Y792C/Y792C^ mice exhibited a larger front limb stride (Supplementary Fig. 2A). Moreover, none of the female *Grm1*^Y792C/Y792C^ mice were able to traverse the wider balance beam without a fall (Supplementary Fig. 2B). These results show that the mGluR1 mutation affects motor coordination in both sexes.

Next, we examined aspects of motor coordination and motor learning in *Grm1* mutant mice using an accelerating Rotarod. No differences in the time taken to fall from the Rotarod, a measure of motor performance, were observed between naive *Grm1* mutant mice and their WT littermates at 3 months of age (Fig. 3D). However, at 12 months of age, *Grm1*^Y792C/Y792C^ mice showed a significantly decreased motor performance compared with *Grm1*^Y792C/+^ and WT animals across all 4 days of testing (Fig. 3D). In contrast, motor learning across consecutive testing days was not affected as mutant animals showed significant improvements over the testing days (Fig. 3D).

Taken together, our results show that mGluR1 GOF results in a dose-dependent and progressive decline of motor coordination and balance in *Grm1* mutant mice, with homozygous *Grm1*^Y792C/Y792C^ mice showing deficits from an early age, and *Grm1*^Y792C/+^ mutants displaying a mild and later-onset phenotype. These findings are consistent with the progressive, late-onset ataxia described in human SCA44 patients that carry heterozygous *GRM1* mutations.^22^

### *Grm1* mutant mice are not impaired in non-cerebellar behaviours

In addition to its role in motor control, mGluR1 signalling has been implicated in other important brain functions including regulating spatial working memory,^30,31^ anxiety-like behaviour,^32,33^ sensorimotor gating^34^ and fear conditioning.^35,36^ We therefore investigated these specific behaviours in *Grm1* mutant mice.

**Figure 4:**
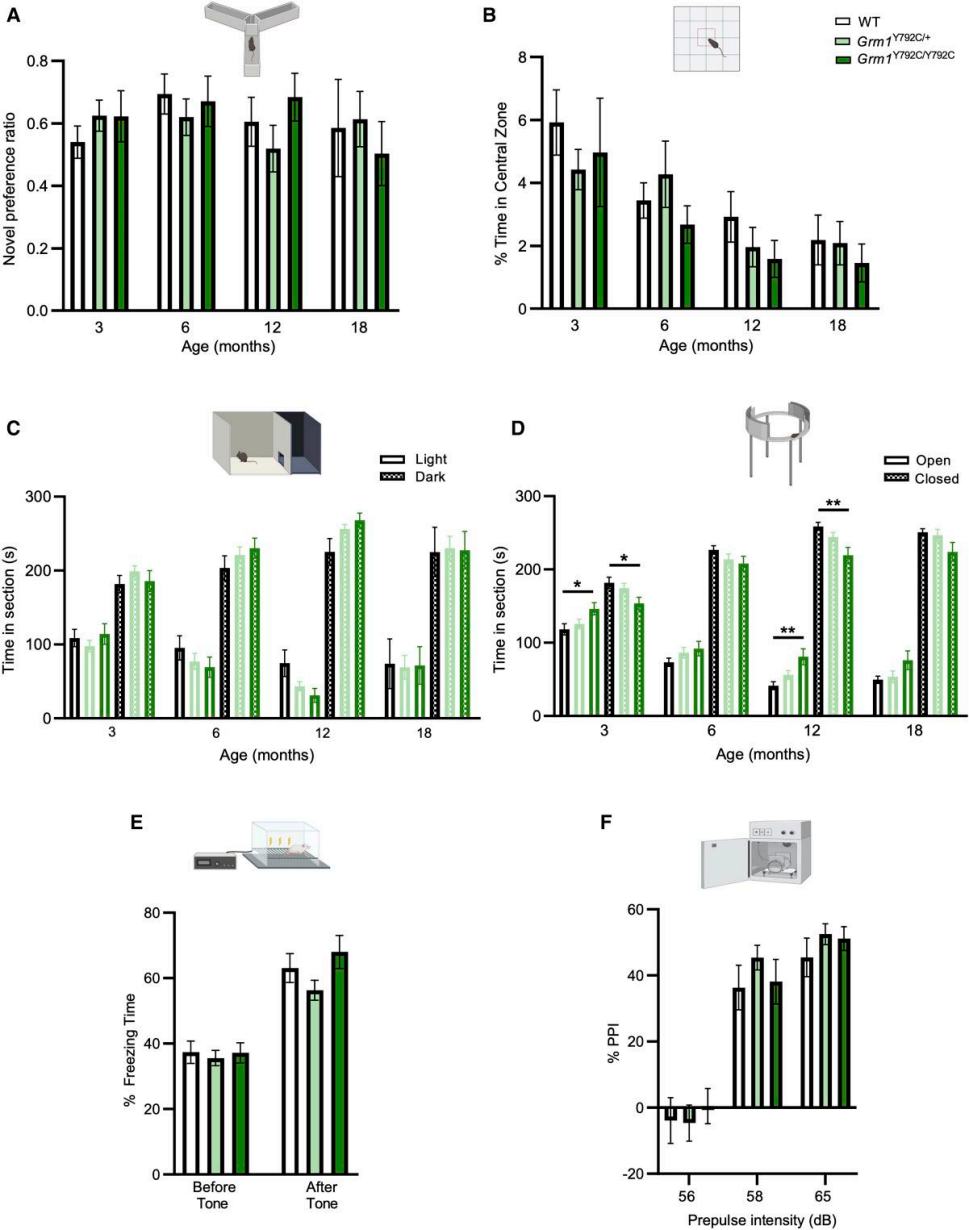
***Grm1* mutant mice do not exhibit deficits in non-motor behaviours**. (A) Time spent by wild-type (WT) and *Grm1* mutant mice in the novel arm on Y-maze compared with the total time spent in the novel and familiar arm expressed as the novel preference arm ratio. (B) Time spent in the central zone in open field assay by WT and *Grm1* mutant mice. (C) Time spent in the light (clear bars) versus dark (filled bars) areas in the light-dark box test by WT and *Grm1* mutant mice. (D) Time spent in the open and closed arms of the zero maze by WT and *Grm1* mutant mice. 3 months: time spent in the open arm, WT versus *Grm1*^Y792C/+^: *P* = 0.7499, WT versus *Grm1*^Y792C/Y792C^: *P* = 0.0436, *Grm1*^Y792C/+^ versus *Grm1*^Y792C/Y792C^: *P* = 0.1221. Time spent in the closed arm, WT versus *Grm1*^Y792C/+^: *P* = 0.7499, WT versus *Grm1*^Y792C/Y792C^: *P* = 0.0436, *Grm1*^Y792C/+^ versus *Grm1*^Y792C/Y792C^: *P* = 0.1221. 12 months: time spent in the open arm, WT versus *Grm1*^Y792C/+^: *P* = 0.3293, WT versus *Grm1*^Y792C/Y792C^: *P =* 0.0095, *Grm1*^Y792C/+^ versus *Grm1*^Y792C/Y792C^: *P* = 0.3104. Time spent in the closed arm, WT versus *Grm1*^Y792C/+^: *P* = 0.3158, WT versus *Grm1*^Y792C/Y792C^: *P* = 0.0091, *Grm1*^Y792C/+^ versus *Grm1*^Y792C/Y792C^: *P* = 0.3157. (E) Per cent freezing during context fear conditioning learning in 6-month-old WT and *Grm1* mutant mice. (**F**) Prepulse inhibition (PPI) values in 6-month-old WT and *Grm1* mutant mice at three different prepulse intensities. Statistical significance was determined by one-way ANOVA followed by Tukey’s *post hoc* test or Kruskal–Wallis test followed by Dunn’s multiple comparison test. For *n*-numbers, see Supplementary Table 1. Error bars represent standard error of the mean. *P* > 0.05 when not indicated. *P < 0.05, **P < 0.01. For full statistical data, see Supplementary Table 2. Behavioural test icons created in BioRender. Becker, E. (2025) https://BioRender.com/um345pp.

We assessed short-term spatial working memory in WT and *Grm1* mutant mice using a Y-maze alternation test. In the initial habituation phase of the trial when only two arms were available, the total distance travelled was similar between all genotypes, indicating similar levels of general activity (Supplementary Fig. 3A). In the test phase, when all arms of the Y-maze were open for exploration, mice of all genotypes spent a similar proportion of their time in the novel arm compared with the total time spent in the novel and familiar arms; this finding was consistent at all four time points tested, suggesting that short-term spatial working memory is unaltered in both *Grm1* mutants (Fig. 4A).

To evaluate *Grm1* mutant mice for anxiety-like behaviour, we applied three independent tests that compare time spent in open versus closed areas of the apparatus. As before, the total distance travelled in these tests was similar between all three genotypes, suggesting that the results from these tests were not influenced by differences in general activity (Supplementary Fig. 3B–D). In the open field and the light/dark box, the percentage of time spent in the open central zone and in the dark zone, an indication of non-anxious behaviour, was similar between the WT and *Grm1* mutant mice (Fig. 4B and C). Some differences were observed between WT and *Grm1*^Y792C/Y792C^ animals in the zero maze (Fig. 4D), but these were inconsistent, with the mutants only at 3 and 12 months spending more time in the open arm and less time in the closed arm than WT. Overall, these results suggest that anxiety-like behaviour is not affected in *Grm1* mutant mice.

In the fear conditioning test, where freezing behaviour is measured as an index of context- specific fear memory, *Grm1* mutant mice exhibited similar freezing levels to WT mice before and after the conditioning tone (Fig. 4E). We also assessed sensorimotor gating by measuring the prepulse inhibition (PPI) of an auditory startle response. PPI levels increased with increasing prepulse intensity as expected, although there were no significant differences between the three genotypes (Fig. 4F). Together, our behavioural results demonstrate that the Y792C mutation in mGluR1 causes behavioural deficits that are linked specifically to motor-and balance-related cerebellar dysfunction, and that enhanced mGluR1 signalling does not significantly affect the other behaviours tested here.

### Structural changes in the *Grm1* mutant cerebellum

**Figure 5:**
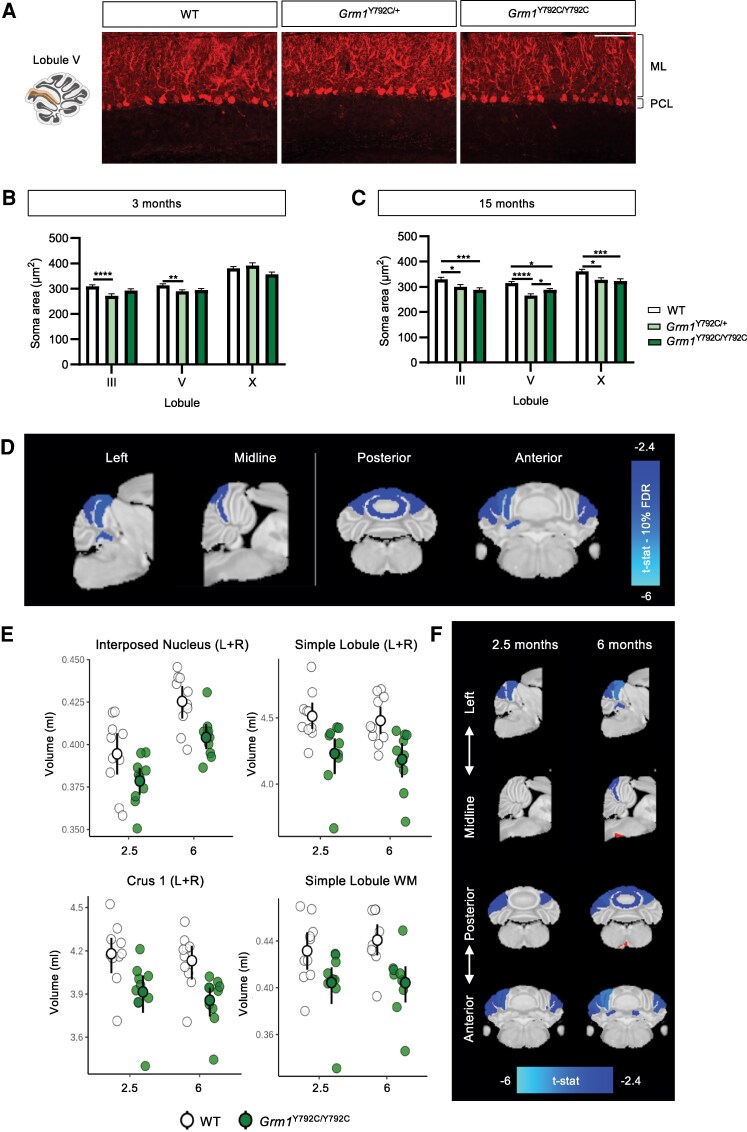
**Grm1 mutant mice exhibit regional cerebellar atrophy.** (A) Representative immunostaining for the Purkinje cell (PC) marker Calbindin in lobule V in 15-month-old wild-type (WT) and *Grm1* mutant cerebellum. ML = molecular layer; PCL = Purkinje cell layer. Scale bar = 100 μm. Cerebellar icons created in BioRender. Becker, E. (2025) https://BioRender.com/um345pp. (B) Quantification of PC soma area in different lobules (III, V and X) in 3-month-old WT and *Grm1* mutant cerebellum. Lobule III: WT versus *Grm1*^Y792C/+^: *P* < 0.0001, WT versus *Grm1*^Y792C/Y792C^: *P* = 0.107, *Grm1*^Y792C/+^ versus *Grm1*^Y792C/Y792C^: *P* = 0.0504; lobule V: WT versus *Grm1*^Y792C/+^: *P* = 0.0093, WT versus *Grm1*^Y792C/Y792C^: *P* = 0.0501, *Grm1*^Y792C/+^ versus *Grm1*^Y792C/Y792C^: *P* > 0.9999; lobule X: WT versus *Grm1*^Y792C/+^: *P* > 0.9999, WT versus *Grm1*^Y792C/Y792C^: *P* = 0.1147, *Grm1*^Y792C/+^ versus *Grm1*^Y792C/Y792C^: *P* = 0.1165. (C) Quantification of PC soma area in different lobules (III, V and X) in 15-month-old WT and *Grm1* mutant cerebellum. Lobule III: WT versus *Grm1*^Y792C/+^: *P* = 0.0364, WT versus *Grm1*^Y792C/Y792C^: *P* = 0.0009, *Grm1*^Y792C/+^ versus *Grm1*^Y792C/Y792C^: *P* = 0.8773; lobule V: WT versus *Grm1*^Y792C/+^: *P* < 0.0001, WT versus *Grm1*^Y792C/Y792C^; *P* = 0.0227, *Grm1*^Y792C/+^ versus *Grm1*^Y792C/Y792C^: *P* = 0.037; lobule X: WT versus *Grm1*^Y792C/+^: *P* = 0.0137, WT versus *Grm1*^Y792C/Y792C^: *P* = 0.0003, *Grm1*^Y792C/+^ versus *Grm1*^Y792C/Y792C^: *P* > 0.9999. *n* = 3–4 animals for each genotype. Statistical significance was determined by one-way ANOVA followed by Bonferroni’s multiple comparison test or Kruskal–Wallis test followed by Dunn’s multiple comparison test. Error bars represent standard error of the mean. *P < 0.05, **P < 0.01, ***P < 0.001, ****P < 0.0001. For full statistical data, see Supplementary Table 2. (D) MRI imaging reveals regional atrophy in *Grm1*^Y792C/Y792C^ animals. (E) Mean bilateral volumetric changes between WT (white) and mutant (green) are detected at 2.5 and 6 months of age for the interposed nucleus, Crus 1, the simple lobule and its associated white matter. (F) Volumetric changes in the mutant cerebellum observed at 6 months already exist at 2.5 months.

Given the observed cerebellar behavioural deficits in *Grm1* mutant mice, we next investigated whether mutant animals display any structural changes in the cerebellum. Gross cerebellar morphology of mutant mice was indistinguishable from WT mice, even at late age (Fig. 5A, Supplementary Fig. 4A and B). To quantitatively assess potential degenerative changes, we performed immunostaining for Calbindin, a marker for PCs, at both early symptomatic (3 months) and late progressive (15 months) disease stage. Measurements were taken across different lobules of the cerebellum (III, V and X), as anterior and posterior cerebellar regions are known to be differentially affected in cerebellar disease models.^37,38^ We found no significant changes in the thickness of the molecular layer or the number of PCs in the *Grm1* mutant cerebellum (Supplementary Fig. 4B–F). A subtle but significant reduction of the PC soma area was found in the *Grm1* mutant cerebellum in the anterior lobules (III and V) at 3 months of age (Fig. 5B). By 15 months, slightly reduced PC soma size was evident across all measured lobules in both mutants (Fig. 5C). These results suggest that enhanced mGluR1 function does not alter gross cerebellar architecture or cause PC loss during the observed time course.

To investigate changes to the cerebellar structure in greater detail, we next employed MRI imaging. We chose to image the *Grm1*^Y792C/Y792C^ animals compared with WT littermates at two different time points, as only the homozygous mutants were showing clear progressive ataxic phenotypes from an early age (Fig. 3). At an early symptomatic stage (2.5 months), we observed regional atrophy in the *Grm1* mutant cerebellum, affecting both the cerebellar cortex and the deep cerebellar nuclei (Fig. 5D and E). In the cerebellar cortex, the affected regions were confined to the lateral hemispheres with significant volume reductions in Crus1 and simplex lobule grey and white matter. Consistent with our histological findings, the atrophy was more pronounced in the anterior cerebellum. The interposed nucleus was also significantly smaller in the mutant mice compared with WT littermates. To evaluate whether the cerebellar atrophy worsened with disease progression, we imaged the mice again at 6 months of age. While the same cerebellar regions remained atrophied, we did not find an increase in the severity of atrophy or additional regions affected (Fig. 5F). However, we cannot rule out that cerebellar atrophy continues over a longer period of time. Together, our findings show that enhanced mGluR1 function results in cerebellar atrophy with anterior regions of the cerebellum affected the most.

### Region- and disease-stage-specific climbing fibre deficits in *Grm1* mutant cerebellum

**Figure 6:**
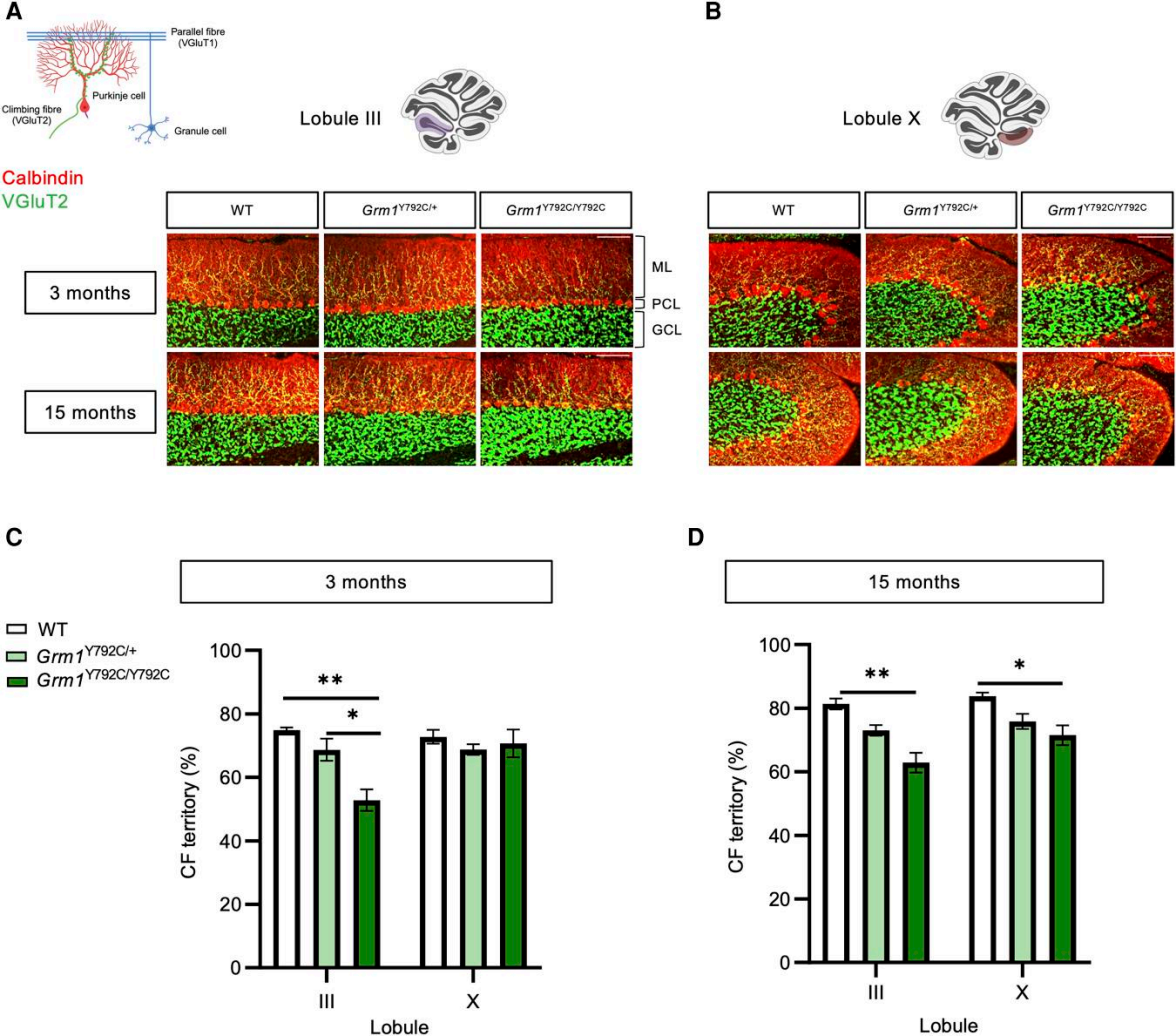
**Lobule- and age-specific reduction in climbing fibre (CF) synapse innervation in *Grm1* mutant cerebellum.** (A and B) Representative immunostaining for the CF synapse marker VGluT2 (green) and the Purkinje cell (PC) marker Calbindin (red) in the anterior lobule III and posterior lobule X from 3-month-old (top) and 15-month-old (bottom) wild-type (WT) and *Grm1* mutant cerebellum. ML = molecular layer; PCL = Purkinje cell layer; GCL = granule cell layer. Scale bar = 100 μm. Cerebellar icons created in BioRender. Becker, E. (2025) https://BioRender.com/um345pp. (C) Quantification of the percentage of the CF territory in lobules III and X in 3-month-old WT and *Grm1* mutant cerebellum. Lobule III: WT versus *Grm1*^Y792C/+^: *P* = 0.4057, WT versus *Grm1*^Y792C/Y792C^: *P* = 0.0018, *Grm1*^Y792C/+^ versus *Grm1*^Y792C/Y792C^: *P* = 0.013; lobule X: WT versus *Grm1*^Y792C/+^: *P* = 0.8761, WT versus *Grm1*^Y792C/Y792C^: *P* > 0.9999, *Grm1*^Y792C/+^ versus *Grm1*^Y792C/Y792C^: *P* > 0.9999. *n* =3–4 animals per genotype. Statistical significance was determined by one-way ANOVA followed by Bonferroni’s multiple comparison test. Error bars represent standard error of the mean (SEM). **P < 0.01. (D) Quantification of the percentage of the CF territory in lobules III and X in 15-month-old WT and *Grm1* mutant cerebellum. Lobule III: WT versus *Grm1*^Y792C/+^: *P* = 0.1920, WT versus *Grm1*^Y792C/Y792C^: *P* = 0.0037, *Grm1*^Y792C/+^ versus *Grm1*^Y792C/Y792C^: *P* = 0.0729; lobule X: WT versus *Grm1*^Y792C/+^: *P* = 0.2424, WT versus *Grm1*^Y792C/Y792C^: *P* = 0.0358, *Grm1*^Y792C/+^ versus *Grm1*^Y792C/Y792C^: *P* = 0.8204. *n* = 3–4 animals per genotype. Statistical significance was determined by one-way ANOVA followed by Bonferroni’s multiple comparison test. Error bars represent SEM. *P < 0.05, **P < 0.01. For full statistical data, see Supplementary Table 2.

mGluR1 signalling is implicated in the spatial organization of PF and CF synapses onto the PCs.^7^ Moreover, altered CF extension along the PC dendritic tree (CF territory) has been observed in several ataxic mouse models including those linked to aberrant mGluR1 signalling.^39,40^ To investigate whether increased mGluR1 activity can directly cause changes in PF and CF innervation, we performed immunostaining for vesicular glutamate transporter 1 (VGluT1), a marker for PF terminals, and VGluT2, a marker for CF terminals in *Grm1* mutant animals at early and late stages of disease. We found that at 3 months of age, the extent of CF territory was significantly reduced in the anterior lobule III in *Grm1*^Y792C/Y792C^ mice compared with WT littermates (Fig. 6A and C). In contrast, CF territory in the posterior lobule X was similar in the WT and *Grm1* mutant cerebellum (Fig. 6B and C). At 15 months of age, CF territory in the *Grm1*^Y792C/Y792C^ cerebellum was significantly reduced in both lobule III and lobule X (Fig. 6A, B and D). No significant reduction in CF territory was observed in either lobule III or X in the *Grm1*^Y792C/+^ cerebellum, even at late stage of disease.

We did not observe any prominent changes in the VGluT1 immunoreactivity or localization in the *Grm1* mutant cerebellum (Supplementary Fig. 5), suggesting that PF-PC synapses remain structurally unaffected by mGluR1 GOF. Together, these results indicate that enhanced mGluR1 signalling causes a progressive reduction in CF innervation that starts in the anterior cerebellum before extending to posterior regions.

### Enhanced mGluR1 activity causes early disruption of Purkinje cell spontaneous activity

**Figure 7:**
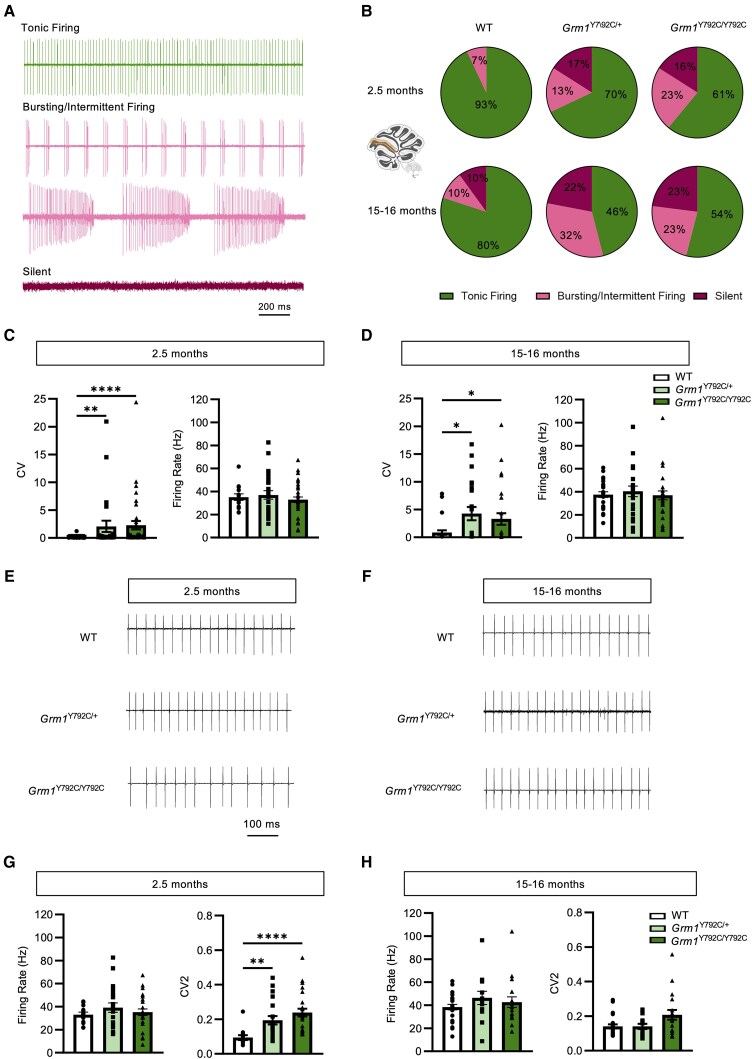
**Spontaneous Purkinje cell firing pattern is disrupted in *Grm1* mutant mice.** (A) Example traces from a tonically firing (green), bursting/intermittently firing (pink) and silent Purkinje cell (PC) (magenta). (B) The percentage of PCs displaying different modes of spontaneous firing activity recorded at 2.5 months (*top*) and 15–16 months of age (*bottom*) in lobule V. WT = wild-type. Cerebellar icon created in BioRender. Becker, E. (2025) https://BioRender.com/um345pp. (C) Quantification of the coefficient of variation (CV) of the interspike interval, and the mean firing rate of all firing PCs at 2.5 months of age. CV: WT versus *Grm1*^Y792C/+^: *P* = 0.0043, WT versus *Grm1*^Y792C/Y792C^: *P* < 0.0001, *Grm1*^Y792C/+^ versus *Grm1*^Y792C/Y792C^: *P =* 0.2895; mean firing rate: WT versus *Grm1*^Y792C/+^: *P* = 0.9262, WT versus *Grm1*^Y792C/Y792C^: *P* = 0.8846, *Grm1*^Y792C/+^ versus *Grm1*^Y792C/Y792^: *P* = 0.5459. *n* = 14 (WT), *n* = 25 (*Grm1*^Y792C/+^), *n* = 37 (*Grm1*^Y792C/Y792C^). (D) Quantification of the CV of the interspike interval, and the mean firing rate of all firing PCs at 15–16 months of age. CV: WT versus *Grm1*^Y792C/+^: *P* = 0.0433, WT versus *Grm1*^Y792C/Y792C^: *P* = 0.0163, *Grm1*^Y792C/+^ versus *Grm1*^Y792C/Y792C^: *P* > 0.9999; mean firing rate: WT versus *Grm1*^Y792C/+^: *P* > 0.9999, WT versus *Grm1*^Y792C/Y792C^: *P* > 0.9999, *Grm1*^Y792C/+^ versus *Grm1*^Y792C/Y792C^: *P* = 0.8172. *n* = 27 (WT), *n* = 22 (*Grm1*^Y792C/+^), *n* = 27 (*Grm1*^Y792C/Y792C^). (**E** and **F**) Example traces of tonically firing PC recordings at 2.5 months (E) and 15–16 months (F). (G) Mean firing rate and precision measured using CV2 of the interspike interval of tonically firing PCs at 2.5 months of age. Mean firing rate: WT versus *Grm1*^Y792C/+^: *P* = 0.4916, WT versus *Grm1*^Y792C/Y792C^: *P* = 0.9120, *Grm1*^Y792C/+^ versus *Grm1*^Y792C/Y792C^: *P* = 0.6348; CV2, WT versus *Grm1*^Y792C/+^: *P* = 0.0036, WT versus *Grm1*^Y792C/Y792C^: *P* < 0.0001, *Grm1*^Y792C/+^ versus *Grm1*^Y792C/Y792C^: *P* = 0.4365. *n* = 13 (WT), *n* = 21 (*Grm1*^Y792C/+^), *n* = 27 (*Grm1*^Y792C/Y792C^). (H) Mean firing rate and precision measured using CV2 of the interspike interval of tonically firing PCs at 15–16 months of age. Mean firing rate: WT versus *Grm1*^Y792C/+^: *P* = 0.6423, WT versus *Grm1*^Y792C/Y792C^: *P* > 0.9999, *Grm1*^Y792C/+^ versus *Grm1*^Y792C/Y792C^: *P* = 0.7577; CV2: WT versus *Grm1*^Y792C/+^: *P* > 0.9999, WT versus *Grm1*^Y792C/Y792C^: *P* = 0.1459, *Grm1*^Y792C/+^ versus *Grm1*^Y792C/Y792C^  *P* = 0.2882. *n* = 24 (WT), *n* = 13 (*Grm1*^Y792C/+^), *n* = 19 (*Grm1*^Y792C/*Y792C*^). Statistical significance was determined by one-way ANOVA followed by Tukey’s multiple comparison test, or Kruskal–Wallis test followed by Dunn’s multiple comparison test. Error bars represent standard error of the mean. *P < 0.05, **P < 0.01, ****P < 0.0001. For full statistical data, see Supplementary Table 2.

PCs fire spontaneous action potentials at high frequencies, and this spontaneous activity is commonly found to be altered in cerebellar diseases.^41^ To investigate whether enhanced mGluR1 activity affects the spontaneous activity of PCs, we performed cell-attached recordings in acute cerebellar slices from *Grm1* mutant mice and WT littermates. We recorded spontaneous activity both at an early disease stage (2.5 months) and at a late time point (15–16 months), when the disease has progressed in *Grm1*^Y792C/Y792C^ mice and subtle motor deficits are present in *Grm1*^Y792C/+^ mice (Fig. 3C). We observed three patterns of PC spontaneous activity: continuous regular tonic firing, bursting or intermittent irregular firing, and silent cells with no firing activity (Fig. 7A).

At the early stage of the disease, the majority of WT PCs displayed tonic firing, while the remaining cells exhibited bursting/intermittent firing. In contrast, the proportion of tonically firing cells was reduced in *Grm1*^Y792C/+^ and *Grm1*^Y792C/Y792C^ mice, respectively (Fig. 7B). An increased proportion of the *Grm1* mutant PCs displayed bursting/intermittent firing activity, which was reflected by a larger number of *Grm1* mutant PCs displaying higher CV values (Fig. 7B and C). The remaining *Grm1* mutant PCs remained silent with no firing activity (Fig. 7B and C). We found no differences in the mean firing rate of all firing cells (tonic plus bursting/intermittent firing) between WT and *Grm1* mutant mice (Fig. 7C).

We made similar observations at late stage of the disease, where the proportion of tonically firing PCs remained lower in *Grm1* mutant mice compared with WT PCs (Fig. 7B). An increased number of mutant PCs displayed bursting/intermittent firing compared with WT, which was reflected by an increased number of mutant PCs displaying higher CV values (Fig. 7B and D). Moreover, we observed an increased percentage of silent PCs in mice from both *Grm1* mutant genotypes. Similar to the early disease stage, the mean firing rate of all firing PCs in *Grm1* mutant mice was similar to WT mice (Fig. 7D).

We next investigated the tonic firing rate and regularity in *Grm1* mutant PCs. At the early disease stage, the mean firing rate of tonically firing PCs was similar in mutant and WT mice (Fig. 7E and G). However, PCs of both *Grm1* mutant mice fired more irregularly and with decreased precision as reflected by a larger CV2 value, which measures the regularity of adjacent interspike intervals (Fig. 7G). However, at the late stage of the disease, the mean firing rate and regularity of tonically firing PCs of *Grm1* mutant mice were similar to WT mice (Fig. 7E and H). These results demonstrate that the spontaneous activity of PCs is disrupted early in both heterozygous and homozygous *Grm1* mutants.

### Lobule-specific variations in disrupted spontaneous activity of Purkinje cells

**Figure 8:**
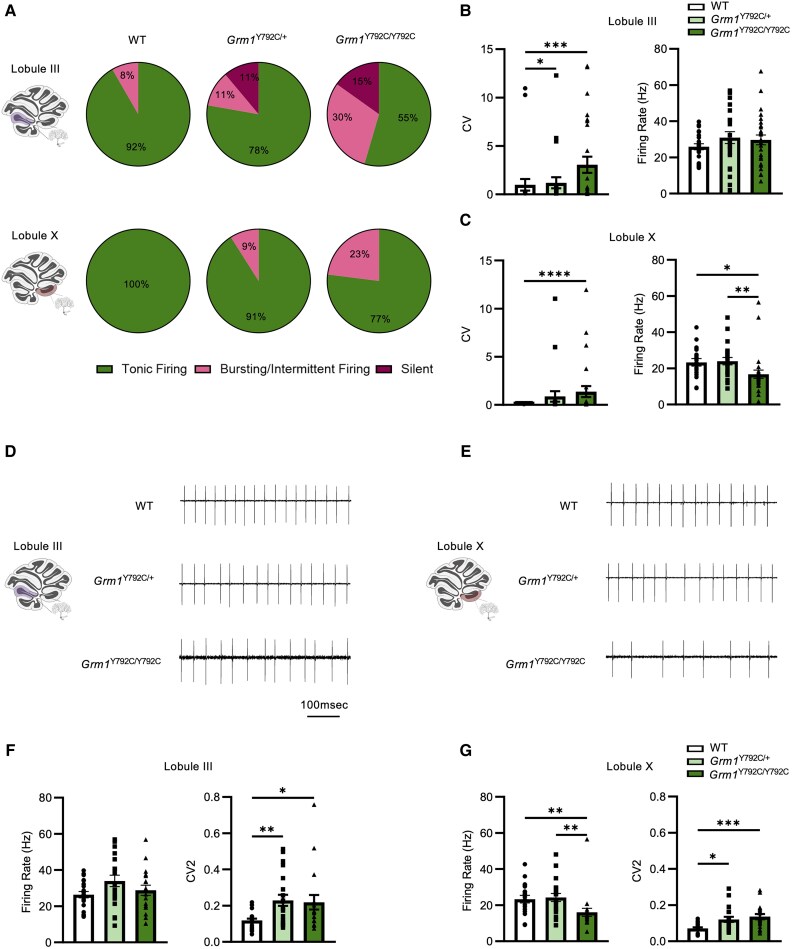
**Lobule-specific disruptions in spontaneous firing patterns of Purkinje cells in *Grm1* mutant mice**. (A) The percentage of Purkinje cells (PCs) displaying different modes of spontaneous firing activity recorded from lobule III (*top*) and lobule X (*bottom*) from 2.5-month-old wild-type (WT), *Grm1^Y792C/+^* and *Grm1^Y792C/Y792C^* mice. (**B**) The coefficient of variation (CV) of the interspike interval, and the mean firing rate of all firing PCs from lobule III. CV: WT versus *Grm1*^Y792C/+^: *P* = 0.0343, WT versus *Grm1*^Y792C/Y792C^: *P* = 0.0009, *Grm1*^Y792C/+^ versus *Grm1*^Y792C/Y792C^: *P* = 0.9458; mean firing rate: WT versus *Grm1*^Y792C/+^: *P* = 0.4041, WT versus *Grm1*^Y792C/Y792C^  *P* = 0.5726, *Grm1*^Y792C/+^ versus *Grm1*^Y792C/Y792C^: *P* = 0.9417. *n* =24 (WT), *n* = 24 (*Grm1*^Y792C/+^), *n* = 28 (*Grm1*^Y792C/Y792C^). **(C)** The coefficient of variation (CV) of the interspike interval, and the mean firing rate of all firing PCs from lobule X. CV: WT versus *Grm1*^Y792C/+^: *P* = 0.0689, WT versus *Grm1*^Y792C/Y792C^: *P* < 0.0001, *Grm1*^Y792C/+^ versus *Grm1*^Y792C/Y792C^: *P* = 0.1224; mean firing rate: WT versus *Grm1*^Y792C/+^: *P* > 0.9999, WT versus *Grm1*^Y792C/Y792C^: *P* = 0.0105, *Grm1*^Y792C/+^ versus *Grm1*^Y792C/Y792C^: *P* = 0.0050. *n* = 19 (WT), *n* = 22 (*Grm1*^Y792C/+^), *n* = 26 (*Grm1*^Y792C/^Y792C). (**D** and **E**) Example traces of tonically firing PC recordings from lobule III (**D**) and lobule X (**E**) of 2.5-month-old WT, *Grm1*^Y792C/+^ and *Grm1*^Y792C/Y792C^ mice. (F) Mean firing rate and precision measured using CV2 of the interspike interval of tonically firing PCs from lobule III. Mean firing rate: WT versus *Grm1*^Y792C/+^: *P* = 0.0932, WT versus *Grm1*^Y792C/Y792C^: *P* = 0.7884, *Grm1*^Y792C/+^ versus *Grm1*^Y792C/Y792C^: *P* = 0.3637; CV2: WT versus *Grm1*^Y792C/+^: *P* = 0.0066, WT versus *Grm1*^Y792C/Y792C^:*P* = 0.0348, *Grm1*^Y792C/+^ versus *Grm1*^Y792C/Y792C^: *P* > 0.9999. *n* = 22 (WT), *n* = 21 (*Grm1*^Y792C/+^), *n* = 18 (*Grm1*^Y792C/^Y792C). (G) Mean firing rate and precision measured using CV2 of the interspike interval of tonically firing PCs from lobule X. Mean firing rate: WT versus *Grm1*^Y792C/+^: *P* > 0.9999, WT versus *Grm1*^Y792C/Y792C^: *P* = 0.0088, *Grm1*^Y792C/+^ versus *Grm1*^Y792C/Y792C^: *P* = 0.0051; CV2: WT versus *Grm1*^Y792C/+^: *P* = 0.0137, WT versus *Grm1*^Y792C/Y792C^: *P* = 0.0007, *Grm1*^Y792C/+^ versus *Grm1*^Y792C/Y792C^: *P* > 0.9999. *n* = 19 (WT), *n* = 20 (*Grm1*^Y792C/+^), *n* = 20 (*Grm1*^Y792C/Y792C^). Statistical significance was determined by one-way ANOVA followed by Tukey’s multiple comparison test, or Kruskal–Wallis test followed by Dunn’s multiple comparison test. Error bars represent standard error of the mean. *P < 0.05, **P < 0.01, ***P < 0.001, ****P < 0.0001. For full statistical data, see Supplementary Table 2. Cerebellar icons created in BioRender. Becker, E. (2025) https://BioRender.com/um345pp.

Given the region-specific vulnerability of PCs in cerebellar ataxia^37,42,43^ and the fact that PC activity differs between cerebellar modules,^8,44^ we next set out to investigate whether enhanced mGluR1 signalling might impact on the spontaneous activity of PCs in a lobule-specific manner. We recorded spontaneous activity of PCs specifically in lobule III and lobule X at 2.5 months of age. mGluR1 protein expression levels were similar across lobules III and X (Supplementary Fig. 6).^45^ As above (Fig. 7), we found that the percentage of tonically firing PCs was reduced in *Grm1* mutant mice, while an increased percentage of mutant PCs exhibited bursting activity and higher CV values (Fig. 8A and B). The remaining mutant PCs were silent. Despite the differences in firing patterns, the mean firing rate across all firing cells (tonic and intermittent firing/bursting cells) did not differ significantly between WT and *Grm1* mutant mice (Fig. 8B).

In lobule X, we also observed an increase in the proportion of bursting PCs in the *Grm1* mutants compared with WT PCs, which were all tonically firing (Fig. 8A and C). In contrast to lobule III, none of the *Grm1* mutant PCs were silent in lobule X. Furthermore, we found that the mean firing rate across all firing PCs was significantly lower in lobule X of the homozygous *Grm1*^Y792C/Y792C^ mice compared with both heterozygous *Grm1*^Y792C/+^ and WT mice (Fig. 8C).

We next examined the properties of tonically firing PCs in lobule III and lobule X. The mean firing rate of tonically firing PCs of lobule III was not significantly different between WT and *Grm1* mutant mice (Fig. 8D and F). In contrast, in lobule X, the mean firing rate of *Grm1*^Y792C/Y792C^ PCs was significantly lower than in WT and *Grm1*^Y792C/+^ (Fig. 8E and G). We found that regardless of lobule location, tonically firing PCs of *Grm1* mutant mice fired more irregularly and with decreased precision, as reflected by the larger CV2 value (Fig. 8F and G).

Together, these findings demonstrate that GOF mGluR1 signalling modulates PC activity in a region-dependent manner that is also dependent on the level of mGluR1 overactivity.

## Discussion

The mGluR1 signalling cascade is critical to the normal functioning of PCs and its disruption results in cerebellar ataxia in humans and animal models. However, the pathophysiological role of aberrant mGluR1 signalling in SCA remains enigmatic as both increase and decrease of mGluR1 function have been linked to cerebellar disease.^4,5^ Increasing evidence points to the presence of enhanced mGluR1 function during early stages of disease. For example, early-stage mouse models of SCA1 and SCA2 display overactive mGluR1 currents.^17,18^ However, changes in other ion channels have also been reported in these models,^46,47^ complicating the interpretation of results. As a consequence, it remains unclear whether increased mGluR1 activity represents a compensatory change or disease-driving mechanism in SCA. In this study, we sought to address the controversy around altered mGluR1 activity by using a mouse model that harbours a GOF mutation in mGluR1, which we had previously identified in SCA44 patients.^22^ We show that *Grm1* mutant mice exhibit cardinal features of SCA, including progressive motor coordination deficits, reduced CF territory and PC firing deficits.^41,48^ These findings demonstrate for the first time that enhanced mGluR1 function in PCs is a direct driver of cerebellar dysfunction and disease.

The p.Y792C mutation was previously shown to behave as a GOF mutation *in vitro*.^22^ Our findings are consistent with this and show enhanced mGluR1-mediated calcium signalling in homozygous *Grm1*^Y792C/Y792C^ PCs and enhanced mGluR1-mediated slow EPSCs in heterozygous *Grm1*^Y792C/+^ PCs. The mechanism behind the divergence in the mGluR1 downstream signalling pathways is unclear. Gene dosage effects might explain the differential functional impact across the two mGluR1 signalling components in *Grm1* mutant mice. Although slow EPSCs observed in *Grm1*^Y792C/Y792C^ PCs appeared larger than WT, these were not found to be statistically significant. This could be due to potential compensatory effects and feedback mechanisms counteracting the excessive mGluR1 activity, which will be interesting to dissect further in the future.

We found that homozygous *Grm1*^Y792C/Y792C^ mutants displayed a more severe motor phenotype with progressive motor deficits manifesting from 3 months of age. Motor deficits in the heterozygous *Grm1*^Y792C/+^ animals were more subtle and only detectable from 12 months onwards on the narrow balance beam. These findings suggest a gene-dosage effect of mutant *Grm1* on phenotypic expression. Homozygous SCA44 patients have not been described, but gene dosage effects and resulting earlier age of onset and more severe clinical phenotypes have been reported in rare homozygous SCA3 and SCA6 patients.^49,50^

mGluR1 is widely expressed in the central nervous system, and loss of mGluR1 function results in behavioural phenotypes that are associated with broader neurological dysfunction including ataxia and impaired motor learning^51^ but also context-dependent deficits in associative learning^52^ and disrupted prepulse inhibition.^34^ Similarly, *GRM1* loss-of-function mutations in humans cause SCAR13,^10,11^ a rare neurodevelopmental disorder characterized by ataxia and intellectual disability, and have also been linked to schizophrenia.^53^ In contrast, in our GOF *Grm1* mutants, we only observed motor phenotypes consistent with cerebellar dysfunction and did not find deficits in any of the non-cerebellar behaviours assessed. These findings suggest that the cerebellum is particularly vulnerable to increased mGluR1 function, which may be due to the lack of compensatory mechanisms that are present in other brain regions. One such compensatory mechanism might be the expression of other group I mGlu receptors; while mGluR1 and mGluR5 are co-expressed in many brain regions where they engage in complex interactions,^54^ their expression in PCs is mutually exclusive.^55^

We identified deficits in spontaneous PC activity in both heterozygous and homozygous *Grm1* mutants at an early age, highlighting the critical role of mGluR1 function in spontaneous PC activity. Moreover, our findings are consistent with the hypothesis that PC firing abnormalities are early drivers of cerebellar dysfunction. We observed a reduction in tonically firing PCs and a concomitant increase in bursting/intermittent firing and silent PCs in *Grm1* mutant mice. Silent PCs are likely depolarized and in a depolarization block.^56,57^ Our findings share important parallels with observations in other mouse mutants in which metabotropic glutamatergic signalling is enhanced. Increased activity of the mGluR1-coupled TRPC3 cation channel has been shown to increase the proportion of silent PCs,^57^ while knockout of the EAAT4 glutamate transporter that maintains low synaptic glutamate levels in PCs results in an increased proportion of bursting PCs.^58^ PKCγ, which acts downstream of mGluR1, reduces large conductance calcium-activated potassium channel currents,^59^ which has been shown to contribute to silent PCs^56^ and firing irregularity in tonically firing PCs.^60^ It will be interesting to further dissect the downstream mechanisms by which enhanced mGluR1 disrupts PC activity and to identify points for potential intervention.

Our study sheds light on the important question of regional vulnerability of PC subpopulations in the cerebellum in disease. Patterned cell death has been observed in a number of cerebellar mouse mutants,^61^ and prominent anterior vermis degeneration occurs in ataxia patients and mouse models of SCA1^37^ and Autosomal Recessive Spastic Ataxia of Charlevoix-Saguenay (ARSACS).^38^ This region-specific vulnerability is thought to be underpinned by distinct molecular identities of PC subpopulations. PCs express aldolase C, also known as zebrin, as well as other molecules in a striped pattern, with anterior lobules containing predominantly zebrin-negative PCs while posterior lobules are enriched in zebrin-positive PCs.^62^ Furthermore, the regional identity of PCs is also associated with functional differences and correlates with differences in firing frequency, intrinsic plasticity and synaptic transmission.^8,44,45,63,64^ Notably, we found that the impact of enhanced mGluR1 activity also manifests in a lobule-dependent manner. Subtle PC soma atrophy and reduced CF innervation were observed early in anterior lobules (III and V) and only later in the posterior lobule X. Moreover, we observed a larger decrease in tonically firing PCs in anterior lobules compared with lobule X, with silent cells detected only in anterior lobules. These results support the interesting hypothesis that anterior PCs are more vulnerable to the pathogenic effects of increased mGluR1 activity. Several factors may contribute to this. Components of the mGluR1 signalling cascade are differentially expressed in different cerebellar lobules. For example, expression of specific mGluR1 receptor isoforms as well as the downstream effector TRPC3 channel are higher in the anterior lobules.^8,45^ Specifically, the short and more active TRPC3c isoform is enriched in the anterior lobules,^45^ likely resulting in larger intracellular calcium levels in zebrin-negative PCs. Glutamate clearance also differs across cerebellar lobules, with the glutamate transporter EAAT4 being highly expressed around zebrin-positive PCs, promoting faster glutamate clearance and limiting mGluR1 activation.^58,65^ This contrasts with the slower glutamate clearance around zebrin-negative PCs due to lower EAAT4 expression, resulting in prolonged mGluR1 activation and likely exacerbating the deleterious effects of hyperactive mGluR1 signalling. These factors could collectively enhance intrinsic excitability and susceptibility of anterior-lobule PCs, resulting in early degenerative changes. The observed decrease in tonic PC firing rate in lobule X at 3 months might also reflect an early adaptive compensatory change and could contribute to the greater resilience of this region. With disease progression in aged animals, the proportion of aberrantly firing cells (silent or bursting) increased, whereas the proportion of tonically firing cells declined. Future work should determine whether this shift reflects progressively impaired mGluR1 function. Compensatory adaptations in *Grm1* mutant mice, together with an age-related loss of firing precision in wild-type animals, may explain the similar firing precision we observed in tonically firing PCs from both genotypes at late disease stages.

Although we identified disease-relevant cellular and behavioural phenotypes in the *Grm1* mutant animals that worsened with age, we did not observe any PC loss. Moreover, the observed disease phenotypes in the heterozygous *Grm1*^Y792C/+^ animals were subtle and differ from the apparent ataxia and cerebellar atrophy reported in heterozygous SCA44 patients.^22^ These observations are similar to findings in other mouse models of SCA, particularly those that express disease mutations at physiological levels and display much subtler phenotypes compared with highly overexpressing transgenic mouse models.^28,66,67^ The discrepancy between human and mouse pathology may relate to the limited lifespan of mice, which may not be long enough to complete the full cascade of pathological changes that follows the initiating event.^68^ Furthermore, human cerebellar PCs exhibit non-allometric structural expansions compared with mice including increased dendritic spine density and PF input, as well as a non-allometric distribution of ion-channel density.^69^ These species differences may render human PCs more vulnerable to excitotoxicity over a lifespan. The more subtle changes detected in the *Grm1* mutant animals, particularly in the heterozygous *Grm1*^Y792C/+^ mice, likely represent early changes during disease. Hence, these animal models may be particularly valuable for investigating early interventions that could prevent further progression of pathology.

Many of the phenotypes we report in the *Grm1* mutant mice including PC firing deficits and CF abnormalities have been demonstrated in other mouse models of SCA including SCA1 and SCA2.^40,41,48^ Together with the studies that report enhanced mGluR1 currents in these models,^17,18^ these findings suggest that enhanced mGluR1 activity represents a common disease mechanism in genetically distinct SCAs. Thus, pharmacological inhibition of mGluR1 presents a promising therapeutic strategy at least for a subgroup of SCAs.

## Supplementary Material

awaf477_Supplementary_Data

## Data Availability

The MRI data that support the findings of this study are available in Zenodo with DOI 10.5281/zenodo.14884600. Code is available on GitHub (https://github.com/clemoune/sca44-memri). All other data are available within the paper and its Supplementary Material.
