# Supplementary material for: Enhanced mGluR1 function causes motor deficits and region-specific Purkinje cell dysfunction
Source: Brain. 2026 Jan 12;149(8):2774–90. doi: 10.1093/brain/awaf477 (PMC13431802; doi:10.1093/brain/awaf477)
Supplement: awaf477_Supplementary_Data [file awaf477_supplementary_data.zip › brain-2025-00673-File010.pdf]

Supplementary Material

Enhanced mGluR1 function causes motor deficits and region-specific Purkinje cell dysfunction

Mohamed F. Ibrahim[1,2], Sevda Boyanova[1,2], Yin Chun Cheng[1,2], Clemence Ligneul[3], Rasneer S. Bains[4], Tiffany C. Johnpulle[1,2], Jason P. Lerch[3,5], Edward O. Mann[6], Peter L. Oliver[7], Esther B. E. Becker[1,2]*

[1]Nuffield Department of Clinical Neurosciences, University of Oxford, Oxford, OX3 9DU, UK
[2]Kavli Institute for Nanoscience Discovery, University of Oxford, Oxford, OX1 3QU, UK
[3]Wellcome Centre for Integrative Neuroimaging, FMRIB, Nuffield Department of Clinical Neurosciences, University of Oxford, Oxford, OX3 9DU, UK
[4]Mary Lyon Centre at MRC Harwell, Didcot, OX11 0RD, UK
[5]Department of Medical Biophysics, University of Toronto, Toronto, ON, M5G 2C4, Canada
[6]Department of Physiology, Anatomy and Genetics, University of Oxford, Oxford, OX1 3PT, UK
[7]Mammalian Genetics Unit, MRC Harwell Institute, Didcot, OX11 0RD, UK

*Corresponding author

Correspondence to: Esther B. E. Becker
Nuffield Department of Clinical Neurosciences
Kavli Institute for Nanoscience Discovery
University of Oxford
Dorothy Hodgkin Crowfoot Building
Sherrington Road
Oxford, OX1 3QU
United Kingdom
Email: esther.becker@ndcn.ox.ac.uk

Supplementary Methods

Generation of *Grm1* mutant mice and genotyping

Grm1 mutant mice were generated under Genome Editing Mice for Medicine (GEMM) programme at the Mary Lyon Centre, Medical Research Council Harwell, UK. Mutant animals were generated by pronuclear injection into one-cell stage embryos (background: C57BL/6NTac) with Cas9 mRNA, single guide mRNA (protospacer sequence: GCAGGTGGTGTACATAGTGA, protospacer adjacent motif (PAM) sequence AGG) and single-stranded oligonucleotide (sequence CTACTATGCCTTCAAGACCCGCAACGTGCCGGCCAATTTCAATGAGGCTAAATACA TCGCgTTCACTATGTgCACCACCTGCATCATCTGGCTGGCTTTTGTTCCCATTTACTT TGGGAGCAACTACAAGAT) in microinjection buffer (10 mM Tris–HCl, 0.1 mM EDTA, 100 mM NaCl, pH7.5). This introduced the SCA44 point mutation (A>C; p.Tyr792Cys) into the endogenous *Grm1* gene as well as an additional silent base pair substitution (C>G; p.Ala782Ala) in the nearby PAM site to prevent re-cutting by the Cas9 enzyme. The MGI ID for the generated allele (*Grm1*^{em1H}) is MGI:6451428.

Routine genotyping was performed from ear tissue samples using PCR (Fwd: 5′-CTCCCATGCCCATTTTGTCC-3′; Rev: 5′-CTTGGGAGTGAACATGCAGC-3′). After amplification, PCR products were digested using FastDigest Bsp1407I restriction enzyme (Thermo Scientific, FD0933), which recognizes a TGTACA restriction site that is only present in the *Grm1* WT allele.

Behavioural testing

MouseWalker gait analysis

Mice were placed and acclimatised to the dark behavioural room at least 30 minutes before the start of experiments. The room was dark, except for the lights of the arena and a computer screen. Each mouse was placed on a raised sealed transparent platform (10 cm *x* ~1 m) lit up by green and red LED light. The walking surface of the arena reflects green LED light except from where the mouse paws touch the glass. The mouse was allowed to run in the apparatus for 1 min while its footsteps were filmed at 81 fps (Basler acA1300-75gc camera) and recorded using the StreamPix 7 software. Videos were analysed and gait parameters were extracted using a custom-trained DeepLabCut[1] model and curated into csv files for analysis using custom-written Python scripts.

Locotronic gait analysis

Paw misplacements during walking were assessed using a Locotronic apparatus (Intelli-bio, Seichamps, France). Mice were challenged to cross a horizontal ladder (bars 3 mm in diameter and 7 mm apart). The crossing was encouraged with a brighter start and a darker zone towards the end of the ladder. Mice were tested for three trials with 30-min interval between each trial. Trials where the mice took longer than 30s to complete the run were excluded from the analysis. The number of front and hind limb slips were analysed.

Balance beam

The apparatus consisted of a beam (~1m long) raised 50 cm above the ground and leading toa dark escape box. Each mouse performed three trials on a wide (25-mm diameter) and narrow (12.5-mm) beam, with a minimum of 10 min rest between trials. At the start of each trial, mice were positioned facing away from the escape box and allowed to turn around on the beam before traversing its length. The number of falls was recorded. Trials where the mouse did not attempt to turn or turned but stayed at the start of the beam for longer than 150s were excluded from the analysis. The percentage of mice traversing the beam without a fall was calculated.

Rotarod

Mouse motor performance was evaluated using an accelerating Rotarod (Ugo Basile, Gemonio, Italy) that accelerated from 4 to 40 rpm over a 5-min period. Mice were tested for three trials each day on four consecutive days and were allowed to rest for 15 min between trials. The trial ended when a mouse either fell from the rod or completed a passive double rotation. The latency to fall or complete passive double rotation was recorded for each trial for each mouse, and the mean value from each of the three trials was calculated to compute the latency to fall for each mouse each day.

Open field test

Open field test was used to assess general locomotor activity and anxiety-like behaviour in a novel environment.[2] Mice were placed in square arenas (40x40 cm) and allowed to freely explore the arena for 20 min. Light levels in the centre of the arena were 150-200 lux, between two and four mice were tested at a time, with one mouse per arena. EthovisionXT tracking software (Noldus, Netherlands) was used to track movements, and to calculate the total distance travelled (in 5-min time bins), and the time spent in the centre of the arena.

Light/dark box test

The light/dark box test was used to assess anxiety-like behaviour. Mice were placed in the light side of the box in the light-dark test apparatus (41(L) x 20(W) x 26(H) cm, length of light section: 25 cm) and allowed to freely explore the apparatus for 5 min. Mouse activity was tracked using Ethovision XT software (Noldus, Netherlands). The time spent and the frequency of entry in the light zone, the dark zone, and the entry zone (*i.e.*, the transition zone between the light and dark zones) were analysed.

Elevated zero maze

The elevated zero maze was used to assess anxiety-like behaviour in the mice. The maze included a circular arena, which was made up of two open sections and two closed sections, with each section about 30 cm long. The mice were placed in an open section and allowed to explore the arena for 5 min. Their activity was tracked using Ethovision XT (Noldus, Netherlands). The time and frequency spent in the open and the closed sections were analysed.

Forced alteration Y-maze

Forced alteration Y-maze was used to test the spatial working memory of the mice. The Y-maze apparatus consisted of three clear Perspex arms (30 x 8 x 20 cm) arranged at 120° angles, and

connected to a central zone The test was divided into two phases: the habituation phase and the test phase. In the habituation phase the mouse was placed in the start arm and allowed to enter the familiar arm, while the entry to the unfamiliar novel arm was blocked. Once the mouse exited the start arm and entered the familiar arm, its activity was recorded for 5 min. Afterwards, the mouse was returned to its home cage for 1 min, while the maze was cleaned. During the test phase, the mouse was returned to the start arm and allowed to explore both the familiar and novel arms for 2 min. The position of the familiar and the novel arm were randomised and balanced between mice to reduce room position (spatial) bias. Animals were tracked using Ethovision XT software (Noldus, Netherlands), and the frequency of arm entries and the total time spent in each arm was analysed. To assess the memory performance of the mice in this test, we calculated novel preference ratio from the test phase of the experiment using the formula:

$$Novel\ preference\ ratio\ = \frac{Time\ in\ novel\ arm\ (s)}{Time\ in\ novel\ arm\ (s) + Time\ in\ familiar\ arm\ (s)} \quad (1)$$

Fear conditioning

This test was used to measure aversive learning and memory in the mice. A neutral conditioned stimulus (CS) – a tone, was paired with an aversive unconditioned stimulus (US) – a mild foot shock. After conditioning, we assessed the spatial context or the CS elicited fear in the absence of US which is shown as lack of movement (freezing). On day 1 – conditioning trial, each mouse was placed in Perspex boxes with metal grid floor and conditioned using a training protocol, consisting of a baseline period, audible tone (20 s), a single foot shock (0.4 mA for 1 s) that co-terminates with the tone, and a period with no stimuli at the end. On day 2 – context trial, each mouse was exposed to the same Perspex box with metal grid floor, but it was not exposed to shocks or tones. After 4 h, the mouse was exposed to a round arena with walls covered in white and black stripes and with Vanillin extract rubbed on the top. This way the mouse was exposed to a visually and olfactory new environment. In this environment, the tone (CS) was played to the mouse. Immobility (freezing) was used as a measure of learning/memory performance.

Prepulse inhibition

Prepulse inhibition of the mice was tested in soundproof boxes. The mice were exposed to initial set of 5 runs with no stimulus, just white noise, and then second set of 5 runs with the startle stimulus only (120 dB). This was followed by 10 of the three types of trials in a pseudorandom order: null stimulus; startle stimulus (0 dB followed by 56 dB; 0 dB followed by 58 dB; 0 dB followed by 68 dB); prepulse and startle stimulus (0 dB followed by 120 dB; 56 dB followed by 120 dB; 58 dB followed by 120 dB; 65 dB followed by 120 dB). The whole run was 45-50 min long. The percentage of prepulse inhibition (PPI%) was calculated for the startle stimulus at each prespulse tone using the formula:

$$PPI\% = 100 - (\frac{Prepulse\ tone}{Startle\ stimulus} x\ 100) \quad (2)$$

RNA extraction and RT-qPCR

Cerebellar total RNA was extracted using the RNeasy Mini kit (Qiagen) according to the manufacturer's instructions and treated with DNase (Qiagen). 1µg of whole cerebellar RNA

was synthesised into cDNA with the SuperScript III First-Strand Synthesis System (Invitrogen). RT-qPCR was performed using the LightCycler 480 SYBR Green master mix (Applied Biosystems, Thermo Scientific), and run on a StepOnePlus™ Real-Time PCR System (Applied Biosystems, Thermo Scientific) under the following reaction conditions: 95°C for 20 min, followed by 40 cycles at 95°C for 3s and 60°C for 30s. Primer sequences: *Grm1* (Fwd: 5'-CGCTCCAACACCTTCCTCAACATT-3', Rev: 5' GGGGTATTGTCCTCTTCCTCCACG-3') and *Gapdh* (Fwd: 5'- TGTGTCCGTCGTGGATCTGA-3', Rev: 5'-TTGCTGTTGAAGTCGCAGGAG-3'). All samples were analysed in triplicate, and *Grm1* expression levels were normalised against the expression levels of *Gapdh* (housekeeping gene) to give relative expression levels ($2^{-\Delta CT}$).

Western blotting

Frozen mouse cerebellar tissue was homogenised in RIPA buffer (Thermo Fisher Scientific) containing cOMPLETE protease inhibitor (Roche) and phosSTOP phosphatase inhibitor cocktail (Roche). Homogenates were incubated for 10 min at 4°C and centrifuged at 15,000 *g* for 30 min at 4°C to obtain protein supernatants. Protein concentration was determined using Pierce™ Bradford Protein Assay Kit (Thermo Fisher Scientific). 30 µg of protein lysate were resolved by SDS-PAGE using 4-12% NuPAGE™ Bis-Tris gel (Invitrogen) at 125V for 100 min and transferred to nitrocellulose membranes (Amersham) at 250 mA for 90 min. Membranes were blocked in 5% milk in TBS-T for 1 h at room temperature. Membranes were then incubated with the following primary antibody (diluted in TBS-T containing 3% BSA) overnight at 4°C: mouse anti-mGluR1α (1:2,500; BD Biosciences; 556389) and rabbit anti-β-actin (1:2,500; Abcam; ab179467). Membranes were washed in TBS-T three times, 10 min each, and incubated with anti-rabbit (1:5,000; Cytiva; NA934V) and anti-mouse (1:5000; Cytiva; NA931V) IgG horseradish peroxidase-conjugated secondary antibodies for 2 h at room temperature, followed by three 10-minute washes in TBS-T. Membranes were developed using Pierce™ ECL Western Blotting Substrate (Thermo Fisher Scientific) on a Bio-Rad ChemiDoc MP Imaging System. Densitometric analyses were performed using FIJI/ImageJ software (version 2.9.0/1.53). Band intensity was normalised to the intensity of β-actin (loading control) on the same membrane to give relative expression levels.

Immunohistochemistry

For 21-month-old mice (Supplementary Fig. 3), paraffin-embedded blocks were used. Coronal cerebellar sections were cut on a microtome at 10 µm and mounted on SuperFrost plus slides. Slides were de-paraffinized with two 5-minute washes in xylene and rehydrated by sequential 2-minute washes with 100%, 95%, 80%, and 70% ethanol. After another two 5-minute washes in PBS, slides were microwaved with antigen retrieval buffer (sodium citrate pH 6.0) for 20 min and allowed to cool for 30 min at RT. Slides were washed with PBS three times, 5 min each, and incubated with blocking buffer (PBS, 5% bovine serum albumin, 0.5% Tween-20) in a humidified chamber for 1 h at RT. After blocking, slides were incubated overnight at 4°C with guinea pig anti-calbindin D28K (1:500; Synaptic Systems; 214005). Slides were washed in blocking buffer three times, 5 min each, and incubated with AlexaFluor 594 goat anti-guinea pig secondary antibody (1:500, Invitrogen, A-11076) for 3 h at room temperature. Slides were

washed in blocking buffer, 5 min each, and then once in PBS for 5 min, before mounting with Vectashield mounting media (Vector Laboratories, H-1200).

Immunostaining of 19-month-old mice (Supplementary Fig. 4) was performed as described in the main manuscript. Cerebellar sections were incubated with rabbit anti-vesicular glutamate transporter 1 (vGluT1) (1:500; Synaptic Systems; 135303) and guinea pig anti-calbindin D28K (1:500; Synaptic Systems; 214005), followed by secondary antibodies AlexaFluor 488 goat anti-rabbit (1:500, Invitrogen, A32731) and AlexaFluor 594 goat anti-guinea pig (1:500, Invitrogen, A-11076).

Immunostaining of 3-4-month-old mice (Supplementary Fig. 6) was performed as described in the main manuscript. Cerebellar sections were incubated with rabbit anti-calbindin D28K (1:10000; Swant; Cb38a) and guinea pig mGluR1α (1:1,000; Nittobo Medical; mGluR1α-GP-Af660), followed by secondary antibodies AlexaFluor 488 goat anti-rabbit (1:500, Invitrogen, A32731) and AlexaFluor 594 goat anti-guinea pig (1:500, Invitrogen, A-11076). Immunofluorescent intensities of mGluR1 in cerebellar sections were quantified using FIJI software by measuring the area and integrated density in the region of interest (molecular layer) for each image. The immunofluorescent intensity was calculated by integrated density − (area of the region of interest × mean fluorescence of background readings). For each image, three background areas without mGluR1 staining were selected to correct for background signals. The intensity was further divided by the area of each region of interest to obtain intensity per μm^2. The values from at least three sections were averaged to obtain the mean immunofluorescent intensity for each animal.

MRI methods

MRI imaging was performed as previously described.[3] *(Acquisitions)* Data were acquired on a 7T Biospec Bruker MRI scanner (Paravision 360.1), equipped with a mouse quadrature cryoprobe. Structural scans were acquired with a multi-gradient-echo sequence (75µm isotropic, TR=50 ms, TEs= [3 6.8 10.6] ms). A manganese chloride solution was used as a contrast agent. Therefore, mice were intraperitoneally injected with a 50 mg/kg solution of $MnCl_2$ 24 hours prior to the scan. Twenty mice (10 *Grm1*$^{Y792C/Y792C}$, 3 females, and 10 WT littermates, 1 female) were scanned at 2.5 and 6 months. Animals were induced with 4% isoflurane in oxygen and anaesthesia was maintained with 1-2% isoflurane in oxygen. Respiration was monitored with a breathing pillow. Temperature was monitored with a rectal probe. Isoflurane level was adapted to keep the animals at 80-110 rpm and a warm water-circulation heating pad was used to keep the animals at 36-37°C during scanning.

(Analysis) For each structural scan, individual echoes were denoised with nonlocal means[4] and averaged together. Individual scans were then co-registered, applying first linear and affine transformations and then a series of nonlinear registrations. Information regarding global deformations (i.e. the overall brain sizes) are stored in these transformation models. The non-linear registration creates a vector field that maps every point in one image to another and provides information about localized deformation (i.e. the relative regional changes). In practice, data were analysed using the MBM Pydpider toolkit[5] with a MAGeT registration for segmentation (MRI atlas).[3,6–10] Group-wise registration was performed on the 40 images (20 animals, 2 time points). Regional volumes were extracted from the individual LSQ6 resampled atlases with the RMINC tools (R package).

For MRI statistical analysis, cerebellar regional volumes were isolated using a hierarchical anatomical tree (RMINC package). A linear mixed effect model was used to assess the effect of age and genotype (fixed effects: age, genotype and sex; random intercept: mouse ID) on cerebellar regional volumes. Multiple comparisons were corrected with a false discovery rate (FDR) threshold of 10%. The interaction between age and genotype was not significant.

Electrophysiology solutions

NMDG aCSF cutting solution (in mM): 92 NMDG, 2.5 KCl, 1.25 NaH_2PO_4, 30 $NaHCO_3$, 20 HEPES, 25 glucose, 2 thiourea, 5 Na-ascorbate, 3 Na-pyruvate, 0.5 $CaCl_2$ and 10 $MgSO_4 \cdot 7H_2O$ with pH titrated to 7.3-7.4 with concentrated HCl.

HEPES aCSF (in mM): 2 NaCl, 2.5 KCl, 1.25 NaH_2PO_4, 30 $NaHCO_3$, 20 HEPES, 25 glucose, 2 thiourea, 5 Na-ascorbate, 3 Na-pyruvate, 2 $CaCl_2$ and 2 $MgSO_4 \cdot 7H_2O$ with pH titrated to 7.3-7.4 with concentrated NaOH.

Sucrose cutting aCSF (in mM): 75 sucrose, 87 NaCl, 2.5 KCl, 1 NaH_2PO4, 25 $NaHCO_3$, 25 glucose, 6 $MgCl_2$, 0.5 $CaCl_2$.

Normal aCSF (in mM): 126 NaCl, 2.5 KCl, 1 NaH_2PO_4, 26 $NaHCO_3$, 2.4 $CaCl_2$, 1.3 $MgCl_2$, 10 glucose.

All reagents were from Sigma-Aldrich.

Supplementary Table 1. Sample size for genotypes used for behavioural analyses at each experimental age. No animals were excluded from the analysis. No criteria were set for inclusion.

	3 Months		6 Months	12 Months	18 Months
	Cohort 1	Cohort 2	Cohort 1	Cohort 1	Cohort 1
**Male**					
Wildtype	15	10	14-15	14	5-8
Grm1$^{Y792C/+}$	23	10	23	23	14-15
Grm1$^{Y792C/Y792C}$	14	10	14	14	11-12
**Female**					
Wildtype	10			11	
Grm1$^{Y792C/+}$	10			11-12	
Grm1$^{Y792C/Y792C}$	10			11	

Supplementary Table 2. Full statistical data (separate .xsl file)

Supplementary Figures

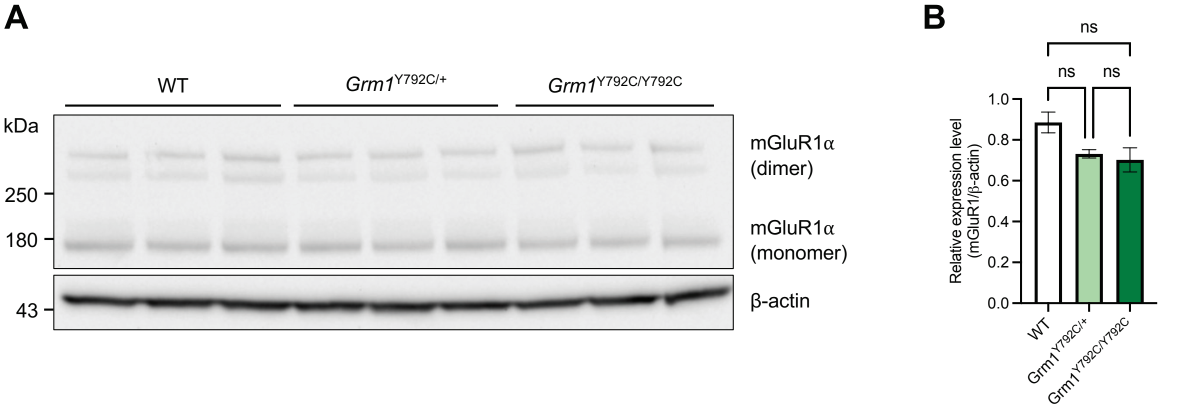

Supplementary Figure 1. mGluR1 protein expression levels in aged *Grm1* mutant mice. **(A)** Representative immunoblotting images for mGluR1 protein expression in 15-to 18-month-old wildtype (WT) and mutant mouse cerebellum. β-actin was used as a loading control. (**B**) Quantification of mGluR1 expression level in the 15- to 18-month-old WT and mutant mouse cerebellum, normalised to the expression levels of β-actin. WT vs. $Grm1^{Y792C/+}$: P=0.1768, WT vs. $Grm1^{Y792C/Y792C}$: P=0.0965, $Grm1^{Y792C/+}$ vs $Grm1^{Y792C/Y792C}$: P>0.9999. n=3 animals per genotype. One-way ANOVA followed by Bonferroni's multiple comparison test. Error bars represent standard error of the mean (SEM). ns, no significance.

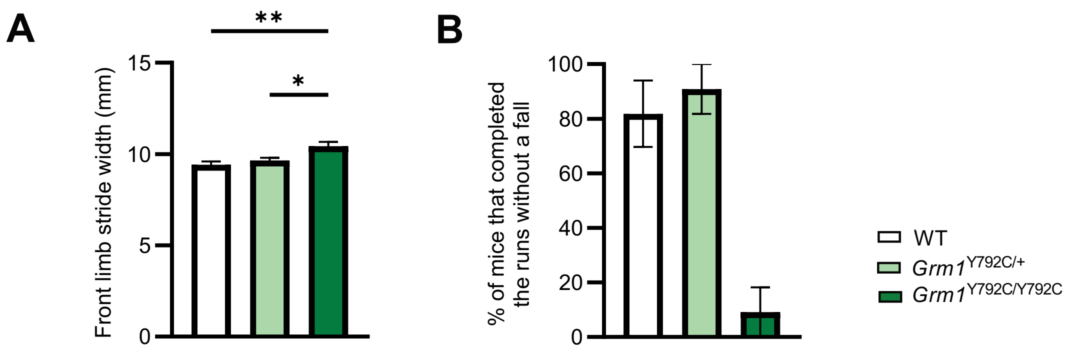

Supplementary Figure 2. Gait and balance are disrupted in naïve 12-month-old female mutant *Grm1*$^{Y792C/Y792C}$ mice. (A) Quantification of the front limb stride width, *i.e.*, the distance between the right and the left front limb placement, assessed using the MouseWalker system. WT vs *Grm1*$^{Y792C/+}$: *P*=0.6905, WT vs *Grm1*$^{Y792C/Y792C}$: *P*=0.0029, *Grm1*$^{Y792C/+}$ vs *Grm1*$^{Y792C/Y792C}$: *P*=0.0231. Statistical significance was determined by one-way ANOVA followed by Tukey's multiple comparison test. For n-numbers, see Supplementary Table 1. **(B)** Quantification of the percentage of mice completing the runs on a wide (25 mm) balance beam without a fall. Error bars represent standard error of the mean (SEM). **P*<0.05, ***P*<0.01, For full statistical data, see Supplementary Table 2. WT = wildtype.

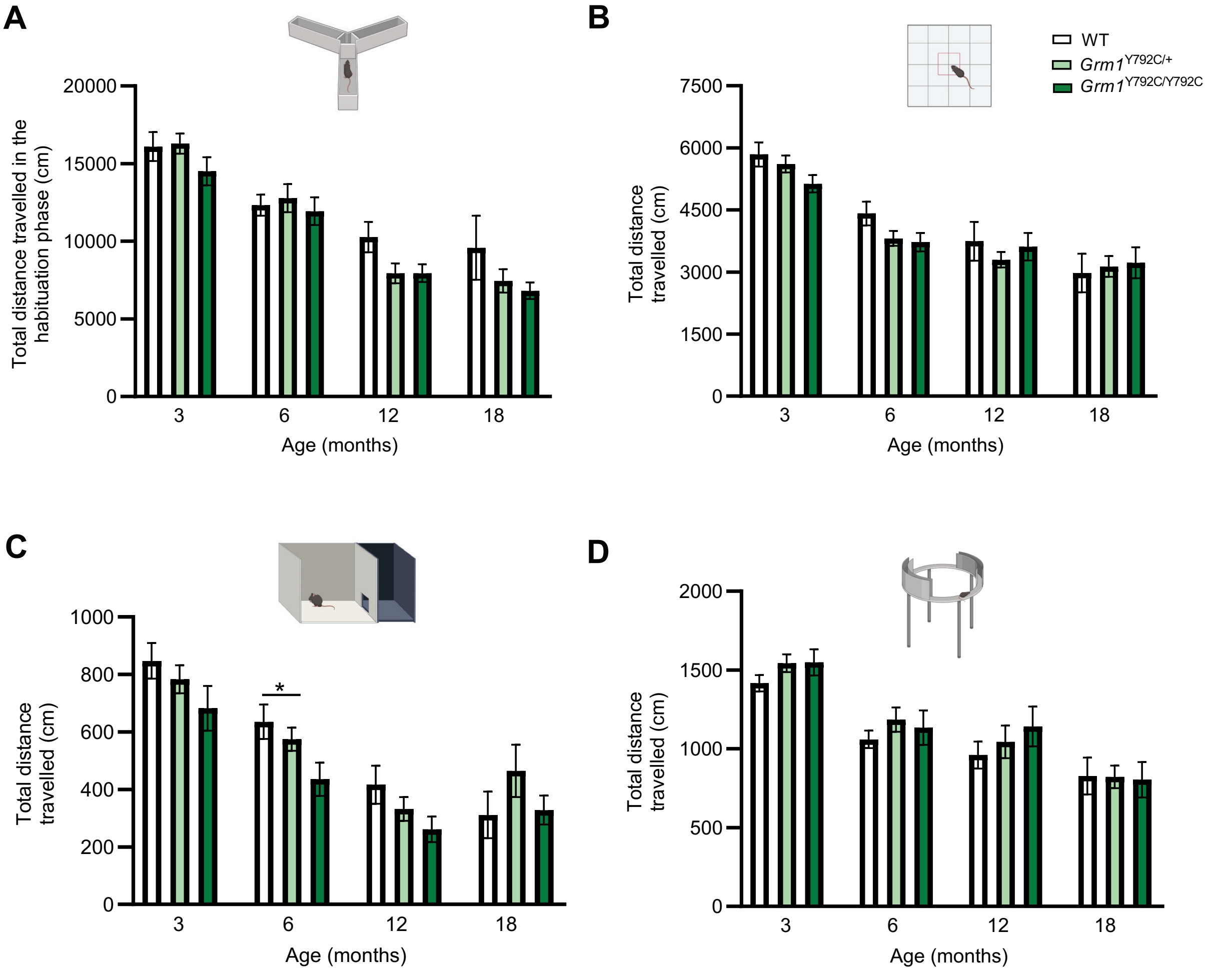

Supplementary Figure 3. *Grm1* mutant mice exhibit no differences in general activity in non-motor behavioural assays. **(A)** Total distance travelled by wildtype (WT) and *Grm1* mutant mice in the habituation phase in the arms on Y-maze. **(B)** Total distance travelled by WT and *Grm1* mutant mice in the open field. **(C)** Total distance travelled by WT and *Grm1* mutant mice in the light/dark box. 6 months: WT vs *Grm1*$^{Y792C/+}$: *P*=0.6649, WT vs *Grm1*$^{Y792C/Y792C}$: *P*=0.037, *Grm1*$^{Y792C/+}$ vs *Grm1*$^{Y792C/Y792C}$: *P*=0.1376. **(D)** Time spent in the central zone in open field assay by WT and *Grm1* mutant mice. Statistical significance was determined by one-way ANOVA followed by Tukey's *post hoc* test or Kruskal-Wallis test followed by Dunn's multiple comparison test. Error bars represent standard error of the mean (SEM). *P*>0.05 when not indicated. **P*<0.05. For full statistical data, see Supplementary Table 2. Behavioural test icons created in BioRender. Becker, E. (2025) https://BioRender.com/um345pp.

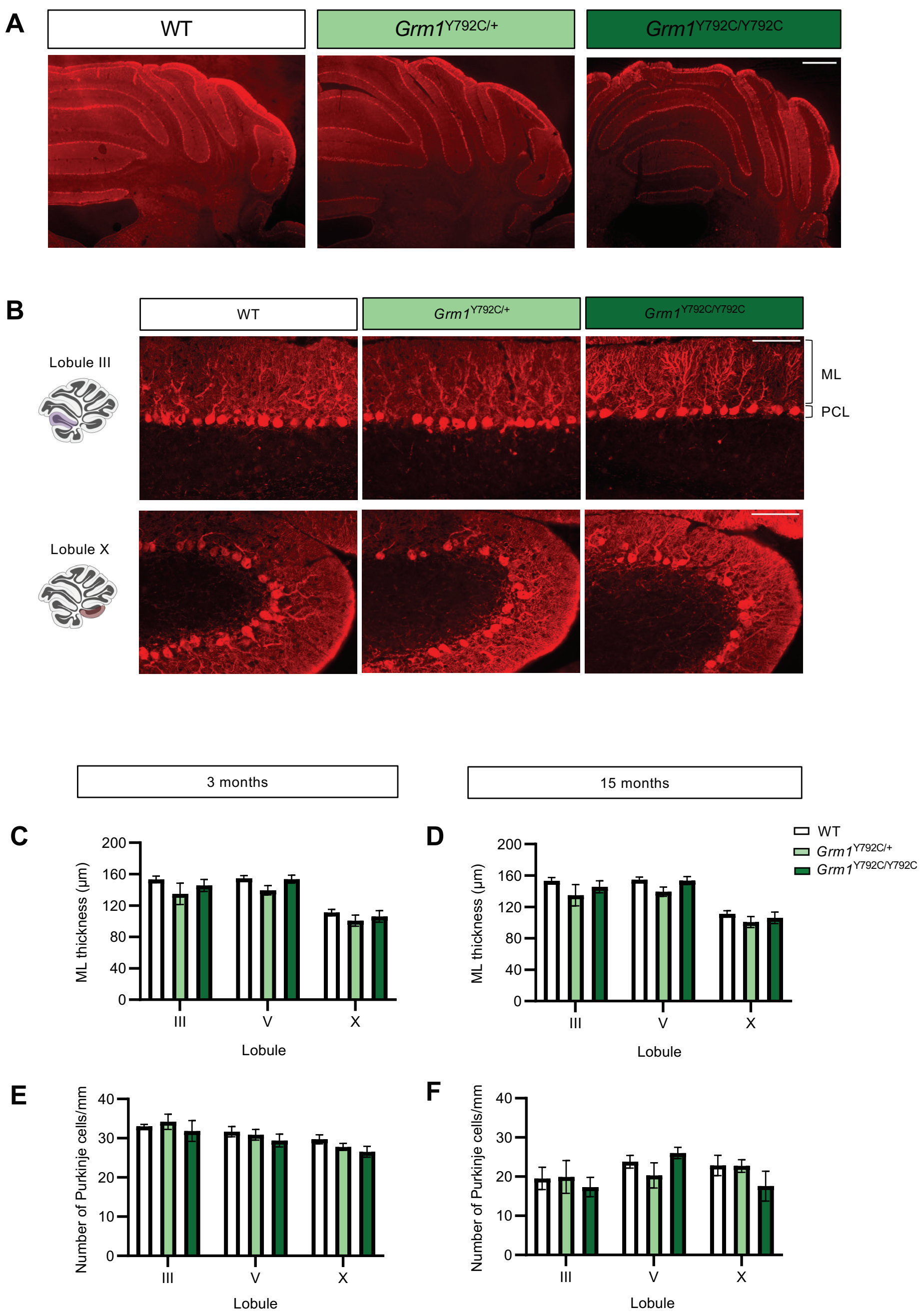

Supplementary Figure 4. No gross changes in cerebellar architecture in wildtype and *Grm1* mutant mice. (A) Representative immunostaining for Purkinje cell (PC) marker Calbindin using coronal sections of 21-month-old wildtype (WT) and *Grm1* mutant cerebellum. Calbindin staining of mutant cerebellum is indistinguishable from WT. Scale bar: 500 µm. **(B)** Representative immunostaining for the PC marker Calbindin in lobules III and X of 15-month-old WT and *Grm1* mutant cerebellum. ML: molecular layer; PCL: Purkinje cell layer. Scale bar: 100 µm. Cerebellar icons created in BioRender. Becker, E. (2025) https://BioRender.com/um345pp. **(C, D)** Quantification of the molecular layer (ML) thickness in different lobules (III, V and X) in three- and 15-month-old WT and *Grm1* mutant cerebellum. No statistical differences are found. **(E, F)** Quantification of PC density in different lobules (III, V and X) in three- and 15-month-old WT and *Grm1* mutant cerebellum. No statistical

differences are found. n=3-4 animals per genotype. One-way ANOVA followed by Bonferroni's multiple comparison test. Error bars represent standard error of the mean (SEM).

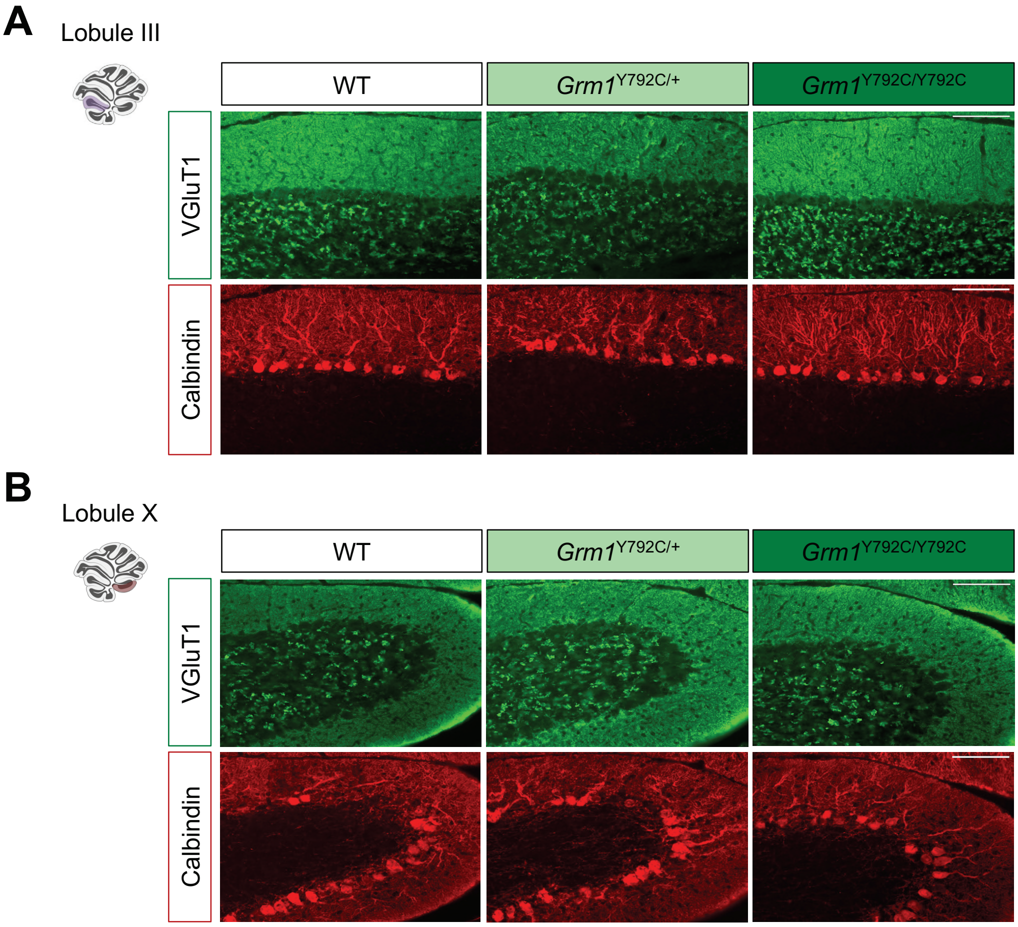

Supplementary Figure 5. No changes in VGluT1 staining in *Grm1* mutant cerebellum. Representative immunostaining for the parallel fibre synapse marker VGluT1 (green) and the Purkinje cell marker Calbindin (red) in lobule III **(A)** and X **(B)** from 15-month-old WT and *Grm1* mutant cerebellum. Scale bar: 100 µm. Cerebellar icons created in BioRender. Becker, E. (2025) https://BioRender.com/um345pp.

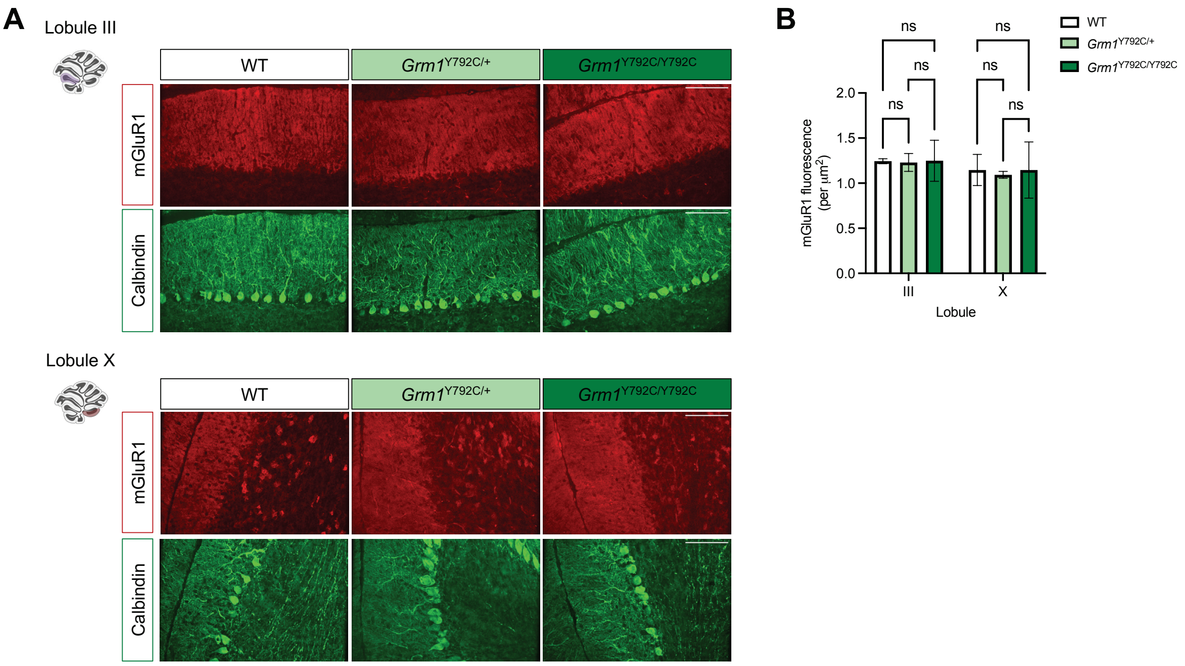

Supplementary Figure 6. mGluR1 protein levels across cerebellar lobules are unchanged in *Grm1* mutant mice. (A) Representative immunostaining for mGluR1 (red) and the PC marker Calbindin (green) in lobule III and X from WT and *Grm1* mutant cerebellum. Scale bar: 100 µm. Cerebellar icons created in BioRender. Becker, E. (2025) https://BioRender.com/um345pp. **(B)** Quantification of mGluR1 immunofluorescence intensity (per μm^2) in the molecular layer from (A). For both lobules: WT vs. *Grm1*$^{Y792C/+}$: P>0.9999, WT vs. *Grm1*$^{Y792C/Y792C}$: P>0.9999, *Grm1*$^{Y792C/+}$ vs *Grm1*$^{Y792C/Y792C}$: P>0.9999. n=3 animals per genotype. One-way ANOVA followed by Bonferroni's multiple comparison test. Error bars represent SEM. ns, no significance.

A

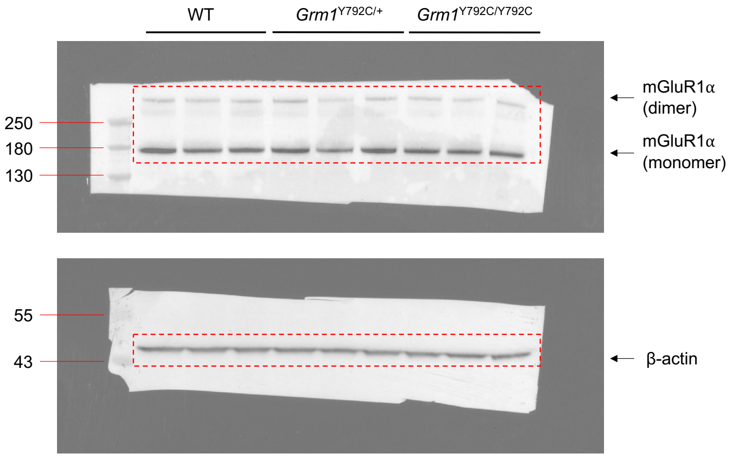

B

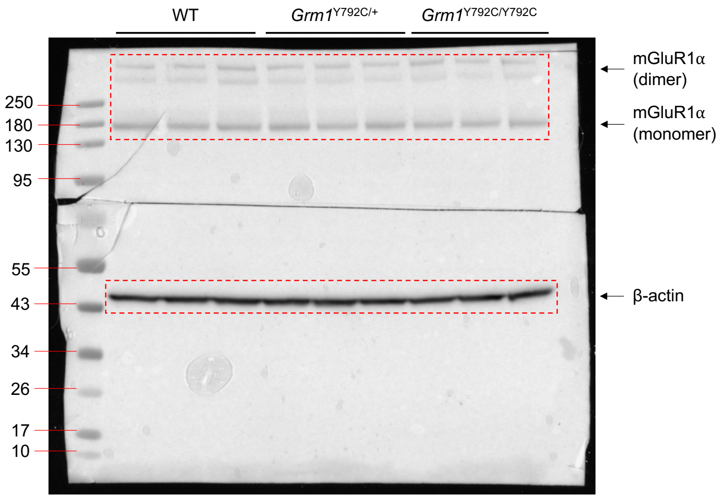

Supplementary Figure 7. Original, uncropped images of Western blots shown in Figure 1E (A) and Supplementary Figure 1A (B). Images are captured using the ChemiDoc Imaging System (Bio-rad). The same membrane is cut into different parts for incubation with primary antibodies against mGluR1α (upper blot) and β-actin as loading control (lower blot). The uncropped images are obtained by merging the colorimetric (for protein ladder) and chemiluminescence (for target protein band) images. The Color Prestained Protein Standard, Broad Range (NEB, P7719S) is used as molecular size marker.

Author Contributions

Conceptualization: P.L.O., E.B.E.B., Methodology: M.F.I., E.M., P.L.O., E.B.E.B., Formal analysis: M.F.I, S.B., Y.C.C., C.L., R.S.B., Investigation: M.F.I, S.B., Y.C.C., C.L., T.C.J., Resources: J.P.L., E.M., P.L.O., E.B.E.B., Writing – Original Draft: M.F.I., E.B.E.B., Writing – Review & Editing: M.F.I, S.B., Y.C.C., C.L., R.S.B., T.C.J., J.P.L., E.M., P.L.O., E.B.E.B., Funding acquisition: J.P.L., P.L.O., E.B.E.B.

Supplementary References

1. Mathis A, Mamidanna P, Cury KM, et al. DeepLabCut: markerless pose estimation of user-defined body parts with deep learning. *Nat Neurosci*. 2018;21(9):1281-1289.

2. Prut L, Belzung C. The open field as a paradigm to measure the effects of drugs on anxiety-like behaviors: a review. *Eur J Pharmacol*. 2003;463(1-3):3-33.

3. Steadman PE, Ellegood J, Szulc KU, et al. Genetic Effects on Cerebellar Structure Across Mouse Models of Autism Using a Magnetic Resonance Imaging Atlas. *Autism Res*. 2014;7(1):124-137.

4. Manjón JV, Coupé P, Martí-Bonmatí L, Collins DL, Robles M. Adaptive non-local means denoising of MR images with spatially varying noise levels. *J Magn Reson Imaging*. 2010;31(1):192-203.

5. Friedel M, Eede MC van, Pipitone J, Chakravarty MM, Lerch JP. Pydpiper: a flexible toolkit for constructing novel registration pipelines. *Front Neuroinformatics*. 2014;8:67.

6. Beera KG, Li YQ, Dazai J, et al. Altered brain morphology after focal radiation reveals impact of off-target effects: implications for white matter development and neurogenesis. *Neuro Oncol*. 2018;20(6):788-798.

7. Dorr AE, Lerch JP, Spring S, Kabani N, Henkelman RM. High resolution three-dimensional brain atlas using an average magnetic resonance image of 40 adult C57Bl/6J mice. *NeuroImage*. 2008;42(1):60-69.

8. Ullmann JFP, Watson C, Janke AL, Kurniawan ND, Reutens DC. A segmentation protocol and MRI atlas of the C57BL/6J mouse neocortex. *NeuroImage*. 2013;78:196-203.

9. Richards K, Watson C, Buckley RF, et al. Segmentation of the mouse hippocampal formation in magnetic resonance images. *NeuroImage*. 2011;58(3):732-740.

10. Qiu LR, Fernandes DJ, Szulc-Lerch KU, et al. Mouse MRI shows brain areas relatively larger in males emerge before those larger in females. *Nat Commun*. 2018;9(1):2615.
